# Modified *N*-acyl-L-homoserine lactone compounds abrogate Las-dependent quorum-sensing response in human pathogen *Pseudomonas aeruginosa*

**DOI:** 10.3389/fmolb.2023.1264773

**Published:** 2023-10-16

**Authors:** Flavio Ballante, Maria V. Turkina, Maria Ntzouni, Karl-Eric Magnusson, Elena Vikström

**Affiliations:** ^1^ Chemical Biology Consortium Sweden (CBCS), Science for Life Laboratory, Department of Medical Biochemistry and Biophysics, Karolinska Institutet, Stockholm, Sweden; ^2^ Department of Biomedical and Clinical Sciences, Faculty of Medicine and Health Sciences, Linköping University, Linköping, Sweden; ^3^ Core Facility, Faculty of Medicine and Health Sciences, Linköping University, Linköping, Sweden

**Keywords:** *Pseudomonas aeruginosa*, quorum sensing, antivirulence strategy, small-molecule probes, *N*-acyl-L-homoserine lactone, LasR, molecular docking, structure-based drug design

## Abstract

Quorum sensing (QS) is a mode of cell–cell communication that bacteria use to sense population density and orchestrate collective behaviors. The common opportunistic human pathogen *Pseudomonas aeruginosa* employs QS to regulate a large set of genes involved in virulence and host–pathogen interactions. The Las circuit positioned on the top of the QS hierarchy in *P. aeruginosa* makes use of *N*-acyl-L-homoserine lactones (AHLs) as signal molecules, like *N*-3-oxo-dodecanoyl-L-homoserine lactone (3O-C_12_-HSL). Disabling QS circuits by certain small-molecule compounds, known as quorum-sensing inhibitors (QSIs), has been proposed as a strategy to attenuate bacterial pathogenicity. In this study, four new AHL analogs were designed by incorporating a tert-butoxycarbonyl Boc group in amide and β-keto (3-oxo) moiety. Compounds were evaluated on a molecular and phenotypic basis as a QSI using the screening strategy linked to the assignment of the Las QS system in *P. aeruginosa*. Using a LasR-based bioreporter, we found that the compounds decreased LasR-controlled light activity and competed efficiently with natural 3O-C_12_-HSL. The compounds reduced the production of the cognate 3O-C_12_-HSL and certain virulence traits, like total protease activity, elastase activity, pyocyanin production, and extracellular DNA release. Furthermore, a quantitative proteomic approach was used to study the effect of the compounds on QS-regulated extracellular proteins. Among the four compounds tested, one of them showed the most significant difference in the appearance of the 3O-C_12_-HSL-responsive reference proteins related to QS communication and virulence, i.e., a distinct activity as a QSI. Moreover, by combining experimental data with computational chemistry, we addressed the effect of LasR protein flexibility on docking precision and assessed the advantage of using a multi-conformational docking procedure for binding mode prediction of LasR modulators. Thus, the four new AHL compounds were tested for their interaction with the AHL-binding site in LasR to identify the key interferences with the activity of LasR. Our study provides further insight into molecular features that are required for small-molecule modulation of LasR-dependent QS communication in *P. aeruginosa*. This should facilitate rational design of the next generation of antivirulence tools to study and manipulate QS-controlled fitness in bacteria and, thereby, handle bacterial infections in a new way.

## Introduction

Bacteria often become tolerant to drugs and antibiotics through the unique features of natural selection, horizontal gene transfer, genetic mutations, and acquisition of mobile genetic elements which have resulted in the spread of resistance associated with the use and overuse of antibacterial agents. Antimicrobial resistance and multidrug resistance, as acquired by several highly virulent human pathogens, pose constant global threats, increasing the challenges to public health. In the last decades, the development and commercialization of novel antibiotics have slowed down or even vanished. Therefore, new antibacterial strategies and agents against antibiotic-resistant pathogens are critically needed (De Oliveira et al., 2020; Mancuso et al., 2021). While the discovery of drugs targeting the growth and viability of bacterial pathogens remains a traditional aim, novel antibacterial approaches to combat bacterial virulence appear more promising. The antivirulence strategy may determine less selective pressure in the development of resistance in contrast to antibiotics (Allen et al., 2014; Roy et al., 2018; Monserrat-Martinez et al., 2019).

The emergency of resistance is associated with the definition of highly virulent superbugs, the so-called ESKAPE pathogens, for which new antimicrobial development is urgently needed. This group comprises *Enterococcus faecium*, *Staphylococcus aureus*, *Klebsiella pneumoniae*, *Acinetobacter baumannii*, *Pseudomonas aeruginosa*, and *Enterobacter* species (De Oliveira et al., 2020; Mancuso et al., 2021).

The Gram-negative bacterium *P. aeruginosa* is ubiquitous in natural environments, especially in locations associated with human activity (Crone et al., 2020). As a human opportunistic pathogen, it is one of the leading causes of nosocomial severe infections located in various host tissues, particularly in patients with impaired immunity and compromised natural barriers. If not properly eradicated during acute illnesses, *P. aeruginosa* can establish chronic conditions that are even more difficult to treat (Pont et al., 2022; Tummler, 2022). This is partly due because bacteria create and live as a biofilm, i.e., a small ecosystem associated with a surface and embedded in a self-producing matrix of macromolecules. This biofilm provides a sustainable community with highly coordinated production of virulent determinants and increased resistance to the immune system and drugs (Hoiby et al., 2010; Breidenstein et al., 2011; Poole, 2011). Moreover, *P. aeruginosa* is equipped with a broad array of virulence traits and sophisticated molecular mechanisms that allow the bacteria to accommodate their lifestyle, manipulate host–pathogen interactions, and escape host defense (Pont et al., 2022; Tummler, 2022). The plasticity of the *P. aeruginosa* genome, supported by global regulatory networks, is a central feature in the pathogen’s ability to adapt and survive within the host. It includes the production of an array of virulent traits, as well as intrinsic, acquired, and adaptive resistance to multiple classes of drugs (Goodman and Lory, 2004; Breidenstein et al., 2011; Moradali et al., 2017). The global regulatory systems of *P. aeruginosa*, like quorum sensing (QS), are considered promising targets for the development of antivirulence drugs (Rutherford and Bassler, 2012). QS is a mode of cell–cell communication employed by bacterial populations based on the production and sensing of signal molecules (Mukherjee and Bassler, 2019). It regulates many aspects of *P. aeruginosa* pathogenic behaviors, such as the timing and production of a plethora of extracellular toxins, degrading enzymes, host defense-inactivating effector proteins, macromolecules in the biofilm matrix, metabolic demands, iron acquisition, and motility (Miranda et al., 2022). New knowledge should give opportunities for the rational design of quorum-sensing inhibitors (QSIs) as the next-generation of antimicrobial agents that are able to reduce rather than compromise bacterial viability. Moreover, this would not disrupt the beneficial host microbiota and would less likely lead to the emergency of resistance (Galloway et al., 2012; Rampioni et al., 2014; Soukarieh et al., 2018).

**FIGURE 1:**
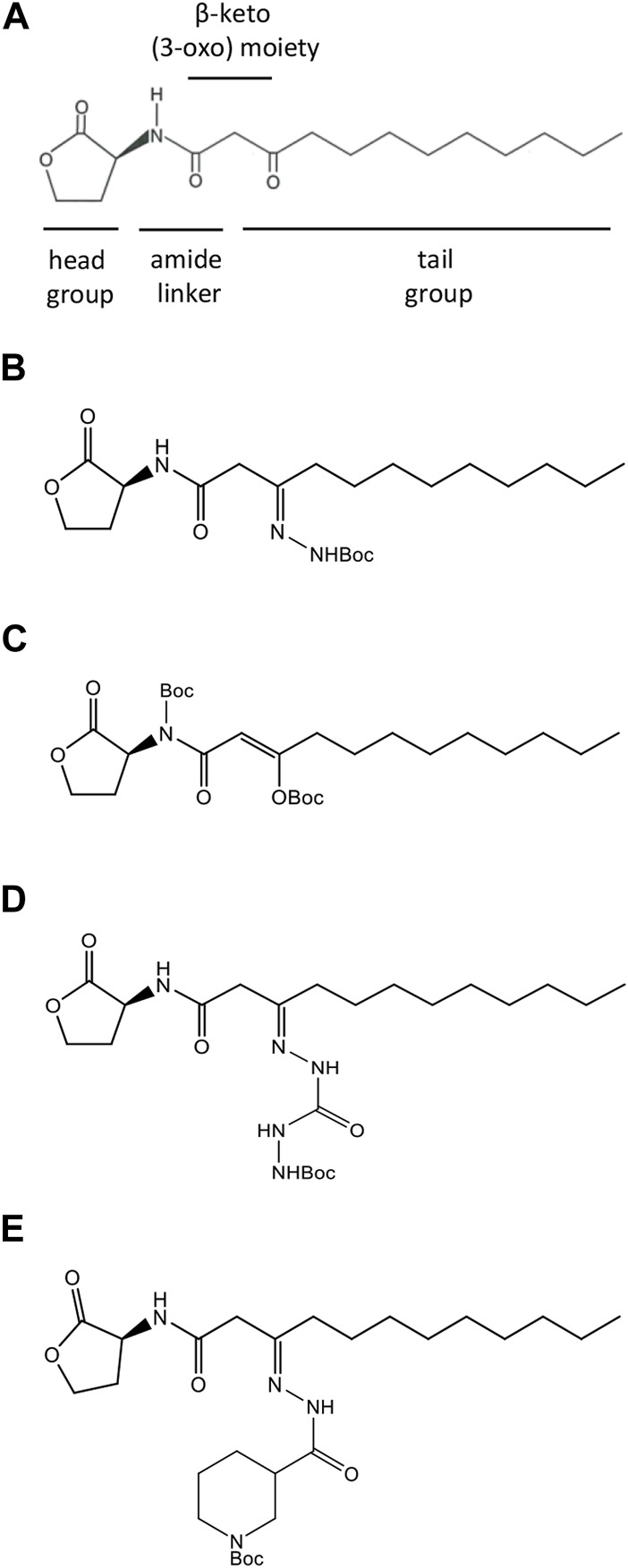
Structures of natural and synthesized AHL molecules. **(A)** Natural *N*-3-oxo-dodecanoyl-L-homoserine lactone, MW 297.4 (3O-C_12_-HSL), employed by *Pseudomonas aeruginosa*. The signature components of AHL include the lactone ring head group, the central amide linker, β-keto (3-oxo) moiety, and tail acyl chain with 12 carbons. (**B–E)** AHL modified by adding tert-butoxycarbonyl Boc groups (AHL compounds). **(B)** N-dodecanoyl-L-homoserine lactone-3-t-butoxycarbonyl hydrazone, MW 411.54 (compound C00). **(C)** N-(t-butoxycarbonyl)-3-((t-butoxycarbonyl)oxy)-dodecanoyl-L-homoserine lactone, E-isomer, MW 497.62 (compound C01). **(D)** N-dodecanoyl-L-homoserine lactone-3-t-butoxycarbonyl hydrazone carbohydrazide, MW 469.57 (compound C03). **(E)** N-dodecanoyl-L-homoserine lactone-3-t-butoxycarbonyl nipecotic acid hydrazone, MW 522.34 (compound C60).

*Pseudomonas aeruginosa* harbors at least four major pathways in its dense network of QS: Las, Rhl, Pqs, and IQS that are interconnected and subordinated with each other, being autoregulatory or controlling the full activity of the others (Papenfort and Bassler, 2016). The tandem Las and Rhl systems rely on a family of diffusible *N*-acyl-L-homoserine lactones (AHLs), while the PQS circuit employs quinolones as signal molecules. In the AHL-based circuits, LasI and RhlI synthases produce the signal *N*-3-oxo-dodecanoyl-L-homoserine lactone (3O-C_12_-HSL, Figure 1A) and *N*-butanoyl-L-homoserine lactone (C_4_-HSL), respectively, which accumulate as bacterial population density increases. At a certain threshold concentration, i.e., a “quorum,” AHL binds to its specific cytoplasmatic receptor which is a transcriptional, regulator protein and activates the expression of multiple genes. The cognate receptor for 3O-C_12_-HSL is LasR, while the receptor of C_4_-HSL is RhlR (Miranda et al., 2022). Together, the QS circuitry in *P. aeruginosa* regulates hundreds of genes (approximately 6% of the whole genome), of which more than 30% encode virulence factors (Schuster and Greenberg, 2006); this results in the differential expression of over 20% of the proteins from major cellular compartments (Arevalo-Ferro et al., 2003; Nouwens et al., 2003). Detection of QS signals *in vitro* and *in vivo* using biosensors can indicate acute or chronic infections, provide a tool to study the interaction between LasR and its potential ligands, and screen for novel antimicrobial quorum-quenching molecules (Massai et al., 2011; Miller and Gilmore, 2020). During the last decades, many investigators have shown that 3O-C_12_-HSL also has distinct impacts on the function and behavior of host cells acting via different mechanisms and signaling pathways (Turkina and Vikstrom, 2019).

A range of small-molecule modulators of QS has been presented, including compounds acting as QSIs by targeting LasR or mimicking AHL and, hereby, attenuating the pathogenicity of *P. aeruginosa*. The Las pathway is generally considered to be the top player in the QS hierarchy, and the LasR receptor is appraised as a key target for QSI development (Galloway et al., 2012; Jakobsen et al., 2013; Soukarieh et al., 2018). QSIs, based on the structure of the natural amphiphilic AHL signaling molecules, bear modifications located in either the lactone ring head group, central amide, or tail lipophilic acyl chain (Figure 1A). Several agonists and antagonists have been discovered when the head group of AHL had been replaced with an aromatic group or bearing cyclic carbocycles (Smith et al., 2003; Marsden et al., 2010). It has also been shown that the tail region of AHL can be incorporated with aromatic functionalities, resulting in analogs with inhibitor activity (Geske et al., 2005; Geske et al., 2008). In addition, either the central amide linker (Frezza et al., 2008; Boukraa et al., 2011; Stevens et al., 2011) or the β-keto (3-oxo) moiety (Forschner-Dancause et al., 2016) represents suitable targets for modifications of AHL, resulting in potential QSI.

Inspired by the previous results in the field, we aimed to perform a rational design of four new AHL analogs in which either amide linker or β-keto (3-oxo) moiety or both were modified by the incorporation of a tert-butoxycarbonyl group (Boc), while the head and tail were retained intact (Figure 1). Despite Boc being a common protective group in organic synthesis, it was used as a modification of a chemotactic peptide agonist to generate an antagonist of formyl peptide receptor signaling in leukocytes (Johansson et al., 1993; Stenfeldt et al., 2007). In addition, a Boc-modified Wnt5a-derived peptide, termed Box5, targeted and antagonized Wnt signaling directly, abolishing melanoma cell activity and representing a candidate for the development of anti-metastatic cancer therapy (Jenei et al., 2009; Yadav et al., 2021). Thus, we hypothesized that Boc modifications in the central connective portions of 3O-C_12_-HSL might result in analogs with anti-QS activity.

Several LasR small-molecule modulators have been reported, paving the way to understand how chemical changes to the main regions of LasR ligands, namely, the head, linker, and tail (Figure 1A), can affect potency and modulation capacity (Soukarieh et al., 2018). Structural biology has provided insight into the binding mode of the LasR activator, highlighting key interactions and how agonists may induce protein conformational changes (e.g., L3 loop) affecting receptor stability (Bottomley et al., 2007; O'Reilly et al., 2018; Paczkowski et al., 2019). Site-directed mutagenesis studies have revealed that interactions with Tyr56, Trp60, and Ser129 drive the effect of LasR modulators (Gerdt et al., 2014). Despite a considerable amount of available data, structure–activity relationships of LasR inhibitors have not been completely elucidated. In addition, the lack of solved protein–inhibitor structures is a limiting factor in understanding how potential antagonists interact with LasR. Consequently, rational design and structure-based approaches for the discovery of LasR antagonists that outcompete the native QS signal remain a challenge.

This work aimed to aid a rational QSI development by designing four new AHL analogs endowed with a tert-butoxycarbonyl group (Boc) and evaluating their efficiency on a molecular and phenotypic basis as QSIs, using a screening strategy linked to the assignment of the Las system in *P. aeruginosa*. Herein, using molecular docking, the binding modes of new compounds to the AHL binding site in LasR were predicted to rationalize key interactions that are likely to be involved in the modulation of the LasR activity. The competition between AHL compounds and 3O-C_12_-HSL was analyzed *in vivo* using a LasR-based biosensor. The biological activity of the compounds was quantified using assays for the production of the cognate 3O-C_12_-HSL and certain virulence traits, like total protease activity, elastase activity, pyocyanin production, and extracellular DNA release. A quantitative proteomic approach was used to study the effect of the compounds on the expression and excretion of QS-regulated extracellular proteins. Overall, the data obtained from our studies provide further insight into molecular features that are required for small-molecule modulation of LasR-dependent QS communication in *P. aeruginosa*.

## Materials and methods

### AHL synthesis

The natural LasR autoinducer, *N*-3-oxo-dodecanoyl-L-homoserine lactone C_16_H_27_NO_4_, MW 297.4 (**3O-C**_**12**_ **-HSL**, Figure 1A), was synthesized by Prof. Peter Konradsson and Lan Bui (Linköping University, Sweden) as described previously (Chhabra et al., 2003). The identity and purity of the synthesized **3O-C**_**12**_ **-HSL** were verified by HPLC, and its activity as a QS molecule was confirmed by the bioassays described earlier (Surette and Bassler, 1998; Winson et al., 1998). Four acyl homoserine lactone compounds (AHL compounds, Figures 1B–E) modified by adding a tert-butoxycarbonyl group (Boc) in different positions, i.e., amide linker and β-keto (3-oxo) moiety, were obtained on request from Cayman Chemical (Ann Arbor, MI) and included the following compounds: N-dodecanoyl-L-homoserine lactone-3-t-butoxycarbonyl hydrazone, MW 411.54 (compound **C00**); N-(t-butoxycarbonyl)-3-((t-butoxycarbonyl)oxy)-dodecanoyl-L-homoserine lactone, E-isomer, MW 497.62 (compound **C01**); N-dodecanoyl-L-homoserine lactone-3-t-butoxycarbonyl hydrazone carbohydrazide, MW 469.57 (compound **C03**); and N-dodecanoyl-L-homoserine lactone-3-t-butoxycarbonyl nipecotic acid hydrazone, MW 522.34 (compound **C60**). For experiments, AHLs were dissolved in 100% acetonitrile as a stock solution and then further diluted in an aqueous buffer or medium of choice.

### Molecular docking

The crystal structures of *P. aeruginosa* LasR, when bound to the natural autoinducer **3O-C**_**12**_ **-HSL**, two analogs of the synthetic activator **TP-1** (originally reported as compounds **17** and **19**), and the synthetic agonist **BB0126** (PDB ID: 2UV0, 6D6O, 6D6P, and 6MWW) (Bottomley et al., 2007; O'Reilly et al., 2018; Paczkowski et al., 2019), were downloaded from the Protein Data Bank (Berman et al., 2000). Following visual inspection, a total of five crystal structure chains were selected (Supplementary Table S4) and prepared for docking calculations as follows: 1) solvent and buffer molecules were removed, 2) hydrogens were added to the complexes, and 3) co-crystallized ligands were finally removed. Re-docking of **3O-C**_**12**_ **-HSL** into its cognate protein structure (PDB ID: 2UV0) was performed by either maintaining or removing a water molecule (ID 2134 in pdb) which has been determined to mediate an H bond between the ligand’s 3-oxo group and Arg61. Ligands were prepared using 1) Marvin to build structures and 2) the cxcalc tool to protonate and generate low-energy conformers (Marvin and cxcalc, version 22.12.0-1538, ChemAxon, https://www.chemaxon.com, see Supplementary Material for the detailed procedure). AutoDockFR v1.2 (Ravindranath et al., 2015) was used to perform docking; PDBQT input files were prepared through the AutoDockFR associate scripts. AutoGridFR was used to generate affinity maps. To account for flexible receptor docking, side chains of Leu36, Tyr47, Ile52, and Arg61 were allowed to rotate. Both rigid and flexible receptor docking were performed using a population of 300 individuals, with a maximum of 25 million energy evaluations and 100 GA evolutions, whereas other parameters were maintained at their default values. Root-mean-squared deviation (RMSD) values against experimental poses were calculated using the *compute_rms_between_conformation.py* function from the AutoDockTools (ADT) package (Morris et al., 2009). Binding modes from AutoDockFR were collected and visually inspected with UCSF Chimera (Pettersen et al., 2004). LigPlot + graphical system was used to generate 2D diagrams of ligand–protein interactions from 3D coordinates (Laskowski and Swindells, 2011).

### Assay for the competition between 3O-C_12_-HSL and AHL compounds using a LasR-based biosensor

The competition between 3O-C_12_-HSL and AHL compounds was assessed using the *P. aeruginosa*-derived biosensor strain PA14-R3, a kind gift from Prof. Giordano Rampioni and Prof. Livia Leoni (University Roma Tre, Italy). The PA14-R3 reporter carries a non-functional allele of the *lasI* gene and is, thus, unable to produce 3O-C_12_-HSL. Still, it harbors the *lasR* gene encoding for the AHL receptor LasR, which perceives the exogenous AHL and responses by inducing bioluminescence (Massai et al., 2011). The addition of a substance with inhibitory activity to the *las*-dependent QS system will reduce light emission by the biosensor. PA14-R3 was routinely grown on Luria-Bertani (LB) agar plates overnight at 37°C. Bacteria were resuspended in the LB medium supplemented with 100 mM MOPS buffer, LB MOPS, pH 7.0 (prepared using a 1 mM stock of 3-morpholinopropane sulfonic acid, sodium acetate trihydrate, and EDTA), to the absorbance at 600 nm wavelength (A600) of 0.18 and further diluted with LB MOPS to an A600 of 0.045. PA14-R3 suspension was aliquoted 100 µL per well in 96-well black plates with clear bottom. A serial dilution of 1:3 of the 3O-C_12_-HSL or the four AHL compounds was set up to obtain 0.005–100 μM final concentrations in bacterial suspensions, and these suspensions were then preincubated for 30 min at room temperature (RT). Then, to assess the competition, bacterial suspensions containing serial dilutions of compounds C00, C01, C03, or C60 were supplemented with 1 μM 3O-C_12_-HSL. Alternatively, bacterial suspensions containing serial dilutions of 3O-C_12_-HSL were supplemented with 1 μM C00, C01, C03, or C60. The mixtures were incubated for 4 h at 37° with gentle shaking. As controls, acetonitrile and LB MOPS were used. Bioluminescence and A600 were measured simultaneously with a plate reader Victor X4 (PerkinElmer, Sweden). The levels of PA14-R3 activity, which is dependent on the biological activity of AHL, were quantified as the ratio between luminescence and A600. At least three independent experiments in triplicates were conducted on separate days and with different bacteria passages.

### *Pseudomonas aeruginosa* PA14 growth, treatment with AHL, and isolation of culture supernatants

The wild-type *P. aeruginosa* strain PA14, originally isolated from human burn wound as UCBPP-PA14 and reported to be highly virulent in diverse host models, e.g., in humans, mice, *Caenorhabditis elegans, Drosophila melanogaster*, and *Arabidopsis thaliana* (Rahme et al., 1995; Mikkelsen et al., 2011), was a kind gift from Prof. Giordano Rampioni and Prof. Livia Leoni (University Roma Tre, Italy). It was routinely cultured on LB agar plates overnight at 37°C. Bacteria were resuspended in a defined minimal medium (Hauser et al., 1998) optimized to produce extracellular proteins, MINS (25 mM KH_2_PO_4_, 95 mM NH_4_Cl, 50 mM C_5_H_8_NO_4_Na monosodium glutamate, and 65 mM C_4_H_4_O_4_Na_2_^.^ 6H_2_O disodium succinate, supplemented with freshly made 5 mM MgSO_4_^.^ 7H_2_O, 18 μM FeSO_4_^.^ 7H_2_O, and 2 mM CaCl_2_^.^ 2H_2_O) to an A600 of 0.1, further diluted with MINS to an initial A600 of 0.015. Bacteria were treated with 50 μM 3O-C_12_-HSL, or compounds C00, C01, C03, or C60, and grown for 4 or 18 h at 37° with shaking and good aeration. As vehicle for AHL, acetonitrile was used. Bacterial growth was quantified in 4-h and 18-h cultures by measuring A600 using a plate reader SpectraMax iD3 (Molecular Devices, San Jose, CA); at least three independent experiments in triplicates were performed on separate days on different bacteria cultures. The 18-h bacterial cultures with a recorded A600 of approximately 1.0 were further processed for the isolation of extracellular protein fraction for proteome analyses and transmission electron microscopy (TEM). Culture supernatants were recovered after 4-h and 18-h incubation by centrifugation at 5,000 g for 20 min at 4°C and then filtered through 0.45 μm syringe filters (Pall Corporation, NY) to remove the bacteria debris. The 4-h culture supernatants were further proceeded for the quantification of 3O-C_12_-HSL production. The 18-h culture supernatants were used in assays for total protease activity, elastase activity, pyocyanin production, and extracellular DNA release.

### Quantification of 3O-C_12_-HSL production

The assay for 3O-C_12_-HSL production by *P. aeruginosa* wild-type PA14 based on biosensor strain PA14-R3 (Massai et al., 2011) was performed as described previously. PA14-R3 was routinely grown on LB agar plates overnight at 37°C, resuspended in LB MOPS to an A600 of 0.18, and further diluted with LB MOPS to an A600 of 0.045. Biosensor suspension was aliquoted 195 µL per well in 96-well black plates with clear bottom. Then, 5 µL of each 4-h culture supernatant was added to the wells containing reporter suspension. As controls, acetonitrile and LB MOPS were used. For the calibration curve, 1:3 serial dilutions of 3O-C_12_-HSL in MINS, with concentrations between 0.005 and 100 μM, were prepared, and 5 µL of each diluted 3O-C_12_-HSL sample was added in the wells with reporter culture. The plates were incubated for 4 h at 37° with gentle shaking. Bioluminescence and A600 were measured simultaneously with a plate reader Victor X4 (PerkinElmer, Sweden). The levels of PA14-R3 activity, which is dependent on the biological activity of AHL, were quantified as the ratio between luminescence and A600. At least three independent experiments in triplicates were performed on separate days and with different bacteria passages.

### Total protease activity

Total protease activity was determined in *P. aeruginosa* PA14 18-h culture supernatants using the EnzChek Protease Assay Kit E6639 (Life Technologies, Grand Island, NY). The analysis is based on casein-derivative substrates labeled with the pH-insensitive red fluorescence dye with almost total quenched conjugate fluorescence. Protease-catalyzed hydrolysis of substrates to peptides results in an increase in fluorescence with an excitation/emission maxima of 568/617 nm, which is proportional to protease activity. All procedures for EnzChek Protease experiments were assayed according to the manufacturer´s recommendations. In brief, 100 µL of the 18-h culture supernatant was added to the casein dye working solution and incubated for 5 h at 37° with gentle shaking in the dark, and the red fluorescence (excitation 550 nm emission 620 nm) was measured using a plate reader SpectraMax iD3. As the supernatants may give the fluorescence background, an additional control without the casein dye substrate was used. The fluorescence background values of the substrate-free controls were subtracted from the fluorescence values of samples containing the substrate and from the MINS blank controls to determine the true fluorescence intensity due to total protease activity. The level of protease activity was calculated by the normalization of the true fluorescence intensity values to the A600 values of the corresponding bacterial culture. At least four independent experiments in triplicates were conducted on separate days and with different bacteria passages.

### Elastase activity

Elastase activity was assessed in *P. aeruginosa* PA14 18-h culture supernatants using Elastin-Congo Red. 1.5 mL tubes were set up, each containing 5 mg of Elastin-Congo Red (Sigma-Aldrich) and 1 mL of protease buffer (100 mM Tris, 1 mM CaCl_2_, pH 7.5). Then, 100 µL of 16-h culture supernatants was added, and suspensions were incubated overnight with shaking at 37°C. They were clarified by centrifugation at 11,000 × g for 10 min at 20°C, and the aqueous phase was separated from the solid pellet. The A495 of the clear phase was measured with a plate reader SpectraMax iD3, using MINS as a blank control, and normalized with respect to the A600 values of the corresponding bacterial culture. At least five independent experiments in triplicates were conducted on separate days and with different bacteria passages.

### Pyocyanin production

Pyocyanin production was determined by measuring the A690 in *P. aeruginosa* PA14 18-h culture supernatants with a plate reader SpectraMax iD3, using MINS as a blank control. The A690 values were normalized to the A600 values of the corresponding bacterial culture and then extrapolated to the calibration curve for purified pyocyanin (Sigma-Aldrich) to calculate the concentration of pyocyanin in culture supernatants. At least four independent experiments in triplicates were conducted on separate days and with different bacteria passages.

### Extracellular DNA release

Quant-it PicoGreen dsDNA reagent (Life Technologies) was used to estimate the quantity of double-stranded DNA (dsDNA). To obtain extracellular nucleic acid-enriched samples, 18-h culture supernatants were mixed with 0.1 volume of 3 M sodium acetate, pH 5.2, and 3 volumes of ice-cold 100% ethanol and then precipitated overnight at −20°C. Pellets were obtained by centrifugation at 13,000 g for 30 min at 20°C, washed twice with 0.5 mL ice-cold 75% ethanol, centrifuged for 10 min each, and then, air-dried and resuspended in TE buffer (1 mM EDTA, 10 mM Tris-Cl, pH 8.0). All procedures for the Quant-it PicoGreen dsDNA assay were performed according to the manufacturer´s recommendations. In brief, a working solution of the PicoGreen reagent in TE buffer was mixed, in equal volume, with the nucleic acid-enriched sample and incubated for 5 min at 20°C in the dark. Green fluorescence (excitation 480 nm emission 520 nm) of the samples was measured with a plate reader SpectraMax iD3 and using TE buffer as a blank control. Fluorescence intensity values were normalized to the A600 values of the corresponding bacterial culture and then extrapolated based on the values obtained for the calibration curve for dsDNA. At least four independent experiments in triplicates were conducted on separate days and with different bacteria passages.

### Isolation of extracellular proteins

*Pseudomonas aeruginosa* PA14 was grown in MINS for 18 h at 37°C with shaking and good aeration, reaching an A600 of approximately 1.0 (late exponential phase–early stationary phase). Then, Halt Protease Inhibitor Cocktail (Thermo Fisher Scientific, Waltham, MA) was added to bacterial cultures. The cells were removed by centrifugation at 5,000 × g for 15 min at 4°C, and supernatants were filtered using 0.45 μm syringe filters (Pall Corporation) to remove bacteria debris. Cell-free supernatants were concentrated using 3 K MWCO 20 mL devices (Pall Corporation) and by centrifugation at 5,000 *g* for 30 min at 17°C. Concentrated samples were washed and desalted in the same devises by adding 10 mL 50 mM ammonium bicarbonate and centrifugation at 5,000 *g* for 20 min at 17°C; this step was repeated three times. Last, the samples were concentrated at 5,000 g for 1 h at 17°C, resulting in a final volume of approximately 1 mL extracellular protein fraction.

### In-solution digestion

The extracellular protein fractions were reduced in 5 mM DTT for 30 min at 60°C and alkylated with 20 mM IAA for 30 min at RT in the dark. DTT was then added again to the samples to a final concentration of 17.5 mM. MS-grade trypsin (Thermo Scientific) was used for the following enzymatic digestion according to the manufacturer’s recommendations. Pierce C18 tips (Thermo Scientific) were used to desalt the obtained peptides. Peptides were then reconstituted in 0.1% formic acid in milliQ water, and peptide concentrations were estimated at A280 (NanoDrop ND-1000 Spectrophotometer, Thermo) prior to liquid chromatography tandem mass spectrometry (LC–MS/MS) analyses.

### Proteomic analysis with LC–MS/MS

Peptides were separated by reverse phase chromatography on a 20 mm × 100 µm C18 pre-column followed by a 100 mm × 75 µm C18 column with particle size 5 µm (NanoSeparations, Nieuwkoop, Netherlands) at a flow rate of 300 nL/min on EASY-nLC II (Thermo Fisher Scientific) by a gradient of 0.1% formic acid in water (A) and 0.1% formic acid in acetonitrile (B) as follows: from 2% B to 30% B in 60 min; from 30% B to 100% B in 60 min. Automated online analyses were performed in a positive mode by using an LTQ Orbitrap Velos Pro hybrid mass spectrometer (Thermo Fisher Scientific) equipped with a nano-electrospray source with Xcalibur software (v.2.6, Thermo Fisher Scientific). Full MS scans were collected with a range of 350–1,800 m/z at resolution of 30,000 (m/z 200), and the top 20 most intense multiple charged ions were selected with an isolation window of 2.0 and fragmented in the linear ion trap by collision-induced dissociation with a normalized collision energy of 30%. Dynamic exclusion was enabled ensuring that peaks selected for fragmentation were excluded for 60 s.

### Proteome database search

Generated raw files were analyzed with Sequest HT in Proteome Discoverer (Thermo Fisher Scientific, San Jose, CA, CS version 1.4.0.288) using the *P. aeruginosa* PA14 protein sequence database available at the NCBI (https://www.ncbi.nlm.nih.gov/protein). Proteins were identified with the following search parameters: trypsin as a digestion enzyme; maximum number of missed cleavages 2; fragment ion mass tolerance 0.50 Da; parent ion mass tolerance 10.0 ppm; carbamidomethylation of cysteine as fixed modification; and methionine oxidation as variable modifications.

### Proteomic data evaluation and label-free quantification

The identified proteins were validated using Scaffold software (Version 4.4.8; Proteome Software Inc., Portland, OR). Identifications were based on a minimum of two peptides, minimum 95% peptide identification probability (using the Scaffold Local FDR algorithm), and minimum 99% protein identification probability using the Protein Prophet algorithm (Nouwens et al., 2003). Proteins containing similar peptides, which could not be differentiated based on LC–MS/MS analysis alone, were grouped to satisfy the principles of parsimony. The label-free quantitative analysis was performed using the total number of spectral counts; normalization was performed to account for variations between samples. Quantitative differences were statistically analyzed by the Fisher exact test using Scaffold software. Differences with *p*-values ≤0.05 were considered statistically significant.

### Protein homology search

Protein sequences were annotated by search in the NCBI database using the Basic Local Alignment Search Tool (BLAST, National Center for Biotechnology Information, Bethesda, MD, United States) with the BLASTP algorithm and UniProt BLAST (EMBI-EBI, Cambridgeshire, United Kingdom) software (https://www.uniprot.org/).

### Transmission electron microscopy

*Pseudomonas aeruginosa* PA14 was grown in MINS for 18 h at 37°C with shaking and good aeration. Bacterial cultures were pre-fixed in an equal volume of 6% glutaraldehyde (Polysciences, Warrington, PA, United States) in 0.1 M Na cacodylate buffer, pH 7.4, for 5 min at 37°C, pelleted by centrifugation at 500 *g* for 10 min at 20°C, and fixed again in 3% glutaraldehyde in 0.1 M Na cacodylate buffer, pH 7.4, for 2 h at RT. The cells were harvested by centrifugation at 500 *g* for 15 min at RT and embedded in 4% gelatin. Gelatin blocks were washed with the same buffer and post-fixed in 1% osmium tetroxide (Polysciences) for 1 h at 4°C. Then, they were rinsed again, *en bloc* stained with 2% uranyl acetate (Polysciences) in 50% ethanol, and dehydrated in a series of ascending concentrations of ethanol and acetone. Prior to embedding in the Durcupan ACM epoxy embedding medium kit (Merck, Sigma-Aldrich), two-step infiltration was performed. Ultrathin sections of 70-nm thickness were prepared using a Leica EM UC7 ultramicrotome (Leica Microsystems GmbH, Vienna, Austria), collected onto formvar-coated slot grids, and counter-stained with uranyl acetate and lead citrate. Height resolution images were taken using a JEM 1400Flash transmission electron microscope (JEOL Ltd., Tokyo, Japan) operated at 80 kV and equipped with a high-speed and -sensitivity XAROSA camera and RADIUS software (EMSIS GmbH, Munster, Germany).

### Statistical analyses

Data in the graphs are presented as mean ± SE, and statistical analyses are based on a paired two-tailed Student’s t-test. *p*-values <0.05 (*), <0.01 (**), and <0.001 (***) were considered significant. This is also specified in the figure legends. For screening and competition assay for AHL, quantification of 3O-C_12_-HSL production, and bacterial growth, at least three independent experiments in triplicates were conducted on separate days on different bacteria passages. For assaying total protease activity and quantification of extracellular DNA and pyocyanin, at least four independent experiments in triplicates were performed on separate days and different bacteria passages. For elastase measurements, at least five independent experiments were conducted in triplicates and on separate days and on different cell passages. The experiments for proteome were repeated at least six times on separate days, and data evaluation, quantification, and statistical analyses are described previously. The TEM experiments were conducted three times on separate days and with different cell passages.

## Results

### Perception of 3O-C_12_-HSL and AHL compounds by LasR

**FIGURE 2:**
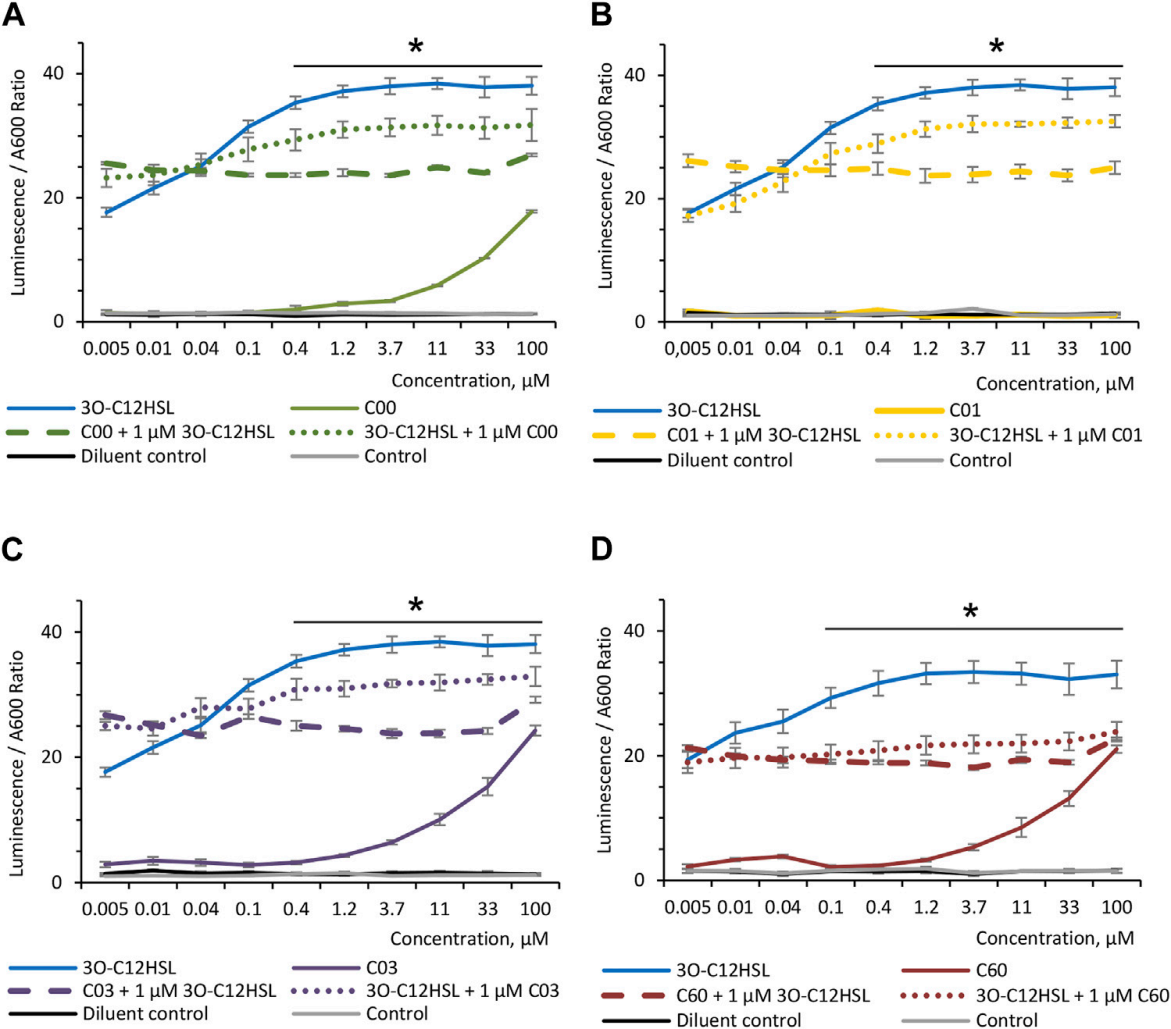
Competition between AHL compounds and 3O-C_12_-HSL for their perception by LasR. Screening was performed for **(A)** compounds C00, **(B)** C01, **(C)** C03, and **(D)** C60 using biosensor PA14-R3. Serial dilution of 1:3 of the 3O-C_12_-HSL (solid blue lines) or the four AHL compounds (solid color-coded lines: green, yellow, lilac, and red) were set up in bacterial suspensions to obtain 0.005–100 μM final concentrations. To assess the competition, serial dilutions of compounds C00, C01, C03, or C60 were set up in bacterial suspensions, preincubated for 30 min at RT, supplemented with 1 μM 3O-C_12_-HSL, and further grown for 4 h at 37° with gentle shaking (dashed color-coded lines). Alternatively, serial dilutions of 3O-C_12_-HSL were set up in bacterial suspensions, preincubated for 30 min at RT, supplemented with 1 μM C00, C01, C03, or C60, and further grown for 4 h at 37° with gentle shaking (dotted color-coded lines). As controls, acetonitrile (diluent control, solid black lines) and LB MOPS (control, solid gray lines) were used. The activities of AHL compounds as a QS signal and their competition with natural 3O-C_12_-HSL for their binding to LasR were accessed *in vivo* using reporter PA14-R3 that responds to endogenous AHL by inducing bioluminescence. Quantification of AHL biological activity and competition was calculated as the ratio between luminescence and A600. At least three independent experiments in triplicates were conducted on separate days and on different bacteria passages. The means ± SE are represented on the curves. Significant differences in comparison to 3O-C_12_-HSL are indicated with * when *p* < 0.05, as analyzed by two-tailed Student’s t-test.

To investigate the ability of AHL compounds and natural 3O-C_12_-HSL to compete with each other, when either of these molecules is bound to LasR, the *P. aeruginosa*-derived biosensor PA14-R3 was used. As expected, when the PA14-R3 reporter was grown in the presence of 0.005–100 μM 3O-C_12_-HSL, the level of its activity was gradually increased, as compared with the control and diluent control (Figure 2). When the PA14-R3 biosensor was cultured in the presence of either compound, the levels of its activity were significantly lower compared to 3O-C_12_-HSL (Figure 2). Remarkably, the activity of the PA14-R3 biosensor remained similar to controls in the presence of compound C01 at all tested concentrations (Figure 2B), suggesting that this compound did not display any agonistic activity. Compounds C00, C03, or C60, when added at low concentrations (ranging between 0.005 and 1.2 μM), also resulted in biosensor activity appearing near the controls. Higher concentrations of compounds C00, C03, or C60 (ranging between 3.7–100 μM) resulted in slightly elevated reporter activity, in contrast to control and diluent control, but still lower compared to 3O-C_12_-HSL (Figures 2A, C, D). It should be noted that AHL molecules at concentrations used in the assay did not alter the growth of the PA14-R3 reporter (not shown). Furthermore, to assess the competition, the biosensor was grown in the presence of 0.005–100 μM C00, C01, C03, or C60 for 30 min and then supplemented with 1 μM 3O-C_12_-HSL. Alternatively, the biosensor was treated with 0.005–100 μM 3O-C_12_-HSL for 30 min and after this supplemented with 1 μM C00, C01, C03, or C60. Here, we detected a significant decrease in the levels of reporter activity, in clear contrast to the levels reached with 3O-C_12_-HSL alone (Figure 2). The addition of 1 μM C00, C01, C03, or C60 after 30-min incubation with 3O-C_12_-HSL was enough to decrease LasR activity when compared to its activity when the reporter was cultured with 3O-C_12_-HSL alone (Figure 2, dotted color-coded lines). Next, the supplement of 1 μM 3O-C_12_-HSL after 30-min incubation with either compound was unable to increase LasR activity in the reporter strain (Figure 2, dashed color-coded lines), reflecting even more efficient competition of compounds with 3O-C_12_-HSL. These results suggest that, likely, the four AHL analogs displayed antagonist activity. To notice, since LasR cannot be purified in its inactive form and in the absence of its natural ligands, e.g., AHL molecules, biosensors are regular tools to study the interaction between LasR and its potential ligands (Massai et al., 2011; Miller and Gilmore, 2020). Taken together, these findings show that the modified AHL compounds can compete with natural 3O-C_12_-HSL for perception by LasR, as they limit the activity of LasR in the biosensor, both alone and in competition with natural 3O-C_12_-HSL.

### Effect of AHL compounds on 3O-C_12_-HSL production in *Pseudomonas aeruginosa*

**FIGURE 3:**
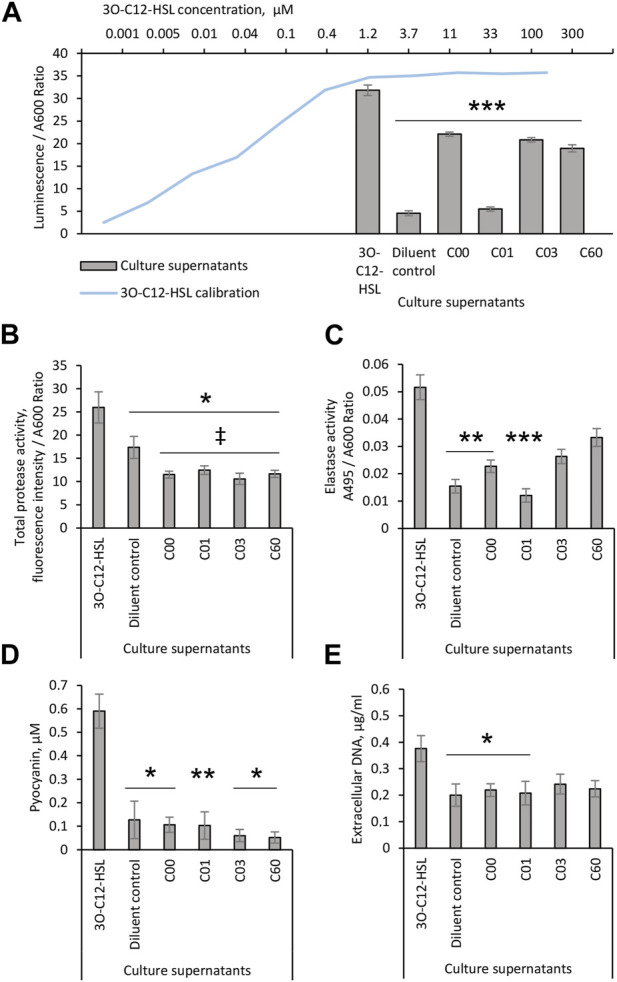
Evaluation of the biological activity of AHL compounds. *Pseudomonas aeruginosa* PA14 cultures were treated with acetonitrile (diluent control) or exposed to 50 μM 3O-C_12_-HSL or compounds C00, C01, C03, or C60. Culture supernatants were recovered after 4-h growth and proceeded for **(A)** analysis of 3O-C_12_-HSL production using reporter PA14-R3. For the calibration curve (blue), 1:3 serial dilutions of 3O-C_12_-HSL, with concentrations between 0.005 and 100 μM, were used. The levels of 3O-C_12_-HSL production were quantified as the ratio between luminescence and A600. At least three independent experiments in triplicates were conducted on separate days and on different bacteria passages. The 18-h culture supernatants of *Pseudomonas aeruginosa* PA14 were evaluated in assays for QS-regulated virulence traits. **(B)** Total protease activity was measured using the EnzCheck Protease fluorescence assay and quantified by normalization of the fluorescence intensity values to the A600 values of the corresponding bacterial culture. At least four independent experiments in triplicates were conducted on separate days and on different bacteria passages. **(C)** Elastase activity was analyzed using Elastin-Congo Red and quantified as the ratio between A495 and A600. At least five independent experiments in triplicates were conducted on separate days and on different bacteria passages. **(D)** Pyocyanin production. The A690 values were normalized to the A600 values of the corresponding bacterial culture and extrapolated to the calibration curve for purified pyocyanin to calculate its concentration in culture supernatants. At least four independent experiments in triplicates were conducted on separate days and on different bacteria passages. **(E)** Extracellular DNA release was measured by Quant-it PicoGreen dsDNA reagent. Fluorescence intensity values were normalized to the A600 values of the corresponding bacterial culture and extrapolated based on the values obtained for the calibration curve for dsDNA. At least four independent experiments in triplicates were conducted on separate days and on different bacteria passages. Columns represent the means ± SE. Significant differences in comparison to 3O-C_12_-HSL are indicated with * when *p* < 0.05 and ** when *p* < 0.01 and *** when *p* < 0.001 as analyzed by two-tailed Student’s t-test. Significant differences in comparison to diluent control are indicated with ‡ when *p* < 0.05 as analyzed by two-tailed Student’s t-test.

Since AHL compounds restricted the LasR activity in the PA14-R3 biosensor, both alone and in competition with 3O-C_12_-HSL, we decided to further validate their ability to block LasR-mediated activity *in vivo* in wild-type *P. aeruginosa*. This was analyzed by the quantification of 3O-C_12_-HSL production in culture supernatants of *P. aeruginosa* PA14 using biosensor PA14-R3 (Figure 3A). We found that the level of 3O-C_12_-HSL in PA14 treated with a diluent was relatively low, yielding approximately the same level as 0.001 µM 3O-C_12_-HSL in calibration controls. As expected, 3O-C_12_-HSL production in PA14 treated with 50 µM 3O-C_12_-HSL was significantly high, in comparison to diluent control. By contrast, the addition of compound C01 to PA14 cultures at 50 µM resulted in significantly lower levels of 3O-C_12_-HSL, similar to the control values. After treatment with 50 μM C00, C03, or C60, we observed a significant decrease in 3O-C_12_-HSL production in PA14, as compared to treatment with 50 µM 3O-C_12_-HSL. It should be noted that 50 µM 3O-C_12_-HSL or AHL molecules did not alter *P. aeruginosa* PA14 growth, as measured after 4-h and 18-h cultivation (Supplementary Figure S1). Together, these data demonstrate that AHL compounds achieve lower production of QS signals, e.g., 3O-C_12_-HSL, *in vivo* in wild-type *P. aeruginosa* PA14.

### Effect of AHL compounds on QS-controlled virulence traits in *Pseudomonas aeruginosa*

It is well established that in *P. aeruginosa*, the QS network including the AHL-dependent network, orchestrates the activation of more than 300 genes in the bacterial genome comprising those that regulate the production of extracellular virulence factors, like elastases, proteases, exotoxin A, and also, exopolysaccharides, rhamnolipids, and extracellular DNA important for biofilm development (Schuster and Greenberg, 2006; Irie and Parsek, 2008). We, thus, further investigated whether the production of QS-controlled virulence traits in wild-type *P. aeruginosa* PA14 was affected by AHL compounds. This was assessed with further quantification of the total protease and elastase activities, pyocyanin production, and extracellular DNA release (Figures 3B–E). Using the EnzCheck Protease fluorescence assay, total protease activity was analyzed and och quantified. We detected significantly lower levels of total protease activity in PA14 growing in the presence of 50 µM AHL compounds, C00, C01, C03, or C60, in clear contrast to either controls or cells treated with 50 µM 3O-C_12_-HSL (Figure 3B). Among many extracellular proteases, the metalloprotease elastase B is a main *las* QS system-regulated virulence factor (Pearson et al., 1997). Therefore, compounds that impair the *las* QS system should be able to inhibit the production of elastase B. Elastase activity was analyzed using Elastin-Congo Red and the quantification of the ratio between A495 and A600. Indeed, we observed that the treatment of PA14 with 50 µM C00 or C01 resulted in significantly lower levels of elastase activity, similar to control. In addition, 50 µM C03 or C60 only tended to reduce the elastase activity (Figure 3C).

In the *P. aeruginosa* QS system, Las/Rhl circuits are deeply interconnected to and subordinated the Pqs circuit affecting together a set of toxic traits and extracellular polymers (Deziel et al., 2004; Lee and Zhang, 2015). Consequently, we analyzed the effect of AHL compounds on the production of the toxic metabolite pyocyanin and the release of extracellular DNA. We detected a low level of pyocyanin in *P. aeruginosa* PA14 cultured in the presence of 50 µM AHL compounds, C00, C01, C03, or C60, similar to that in control and lower than after 3O-C_12_-HSL treatment (Figure 3D). Using the Quant-it PicoGreen dsDNA reagent, AHL compounds were tested for their ability to reduce DNA release in the *P. aeruginosa* cultures. A larger amount of extracellular DNA was detected in culture supernatants of P14 cultivated with 50 µM 3O-C_12_-HSL, in comparison to control. The addition of compound C00 or C01, in contrast to C03 or C60, significantly abolished extracellular DNA production, matching the levels of control (Figure 3E).

It should be noted that 50 µM 3O-C_12_-HSL or the AHL compounds did not alter the planktonic growth (Supplementary Figure S1) and ultrastructure characteristics (Supplementary Figure S2) of the bacteria, which clearly showed a plasma membrane, cell wall, outer membrane, and lipopolysaccharide around the bacteria. The cytoplasm and cytoplasmic components including the nucleoid in the bacteria could be distinguished, respectively, as less electron-dense (lighter) and comparatively higher electron-dense (darker) regions. Additional structures were observed between cells, resembling extracellular polymers or cell debris. Together, these data indicate that AHL compounds, especially C01 and C00, were efficient in inhibiting the activity or production of several extracellular QS-controlled traits, e.g., proteases, elastases, pyocyanin, and DNA, leaving the cell structure seemly unaffected.

### Alterations in the extracellular proteome in response to AHL compounds

Mass spectrometry (MS)-based quantitative proteome profiling is a well-established modern approach to gain insights on molecular mechanisms involved in the pathogenicity and virulence of *P. aeruginosa* (Sauvage and Hardouin, 2020). Since important QS-regulated virulence traits comprise those being secreted to the extracellular milieu, compounds that impair the *las* QS system might affect the expression and excretion of extracellular proteins. To identify differentially expressed proteins in the extracellular space of *P. aeruginosa* PA14 as a response to AHL compounds in comparison to natural 3O-C_12_-HSL, we performed LC–MS/MS-based quantitative analyses of the extracellular protein fractions obtained from *P. aeruginosa* PA14, after being exposed to 50 µM 3O-C_12_-HSL, C00, C01, C03, or C60 or treated with acetonitrile as a diluent control. The bacteria were harvested, and extracellular proteins were collected in a late exponential–early stationary phase where most of the QS-regulated proteins are induced (Arevalo-Ferro et al., 2003; Scott et al., 2013; Sauvage and Hardouin, 2020). More than 1,000 proteins and protein clusters were identified as extracellular according to NCBI GO annotations. Well-known exoproteins, e.g., elastase LasB and protein hcp1, were detected as the most abundant in all samples. The identified proteins were mainly associated with the secreted, outer membrane, periplasm, plasma membrane, cytoplasm, and protein secretion system, but they were also associated with an unknown compartment. It should be noted that many proteins have not yet been annotated as secreted for several reasons: experimental evidence is missing; the translocation system for these proteins is still not well studied; uncertain predictions for their localization. Moreover, approximately 40% of the *P. aeruginosa* proteins are annotated as hypothetical or not yet studied (Armengaud et al., 2013; Ball et al., 2016).

Next, we identified proteins exhibiting significant changes after treatment with 50 µM 3O-C_12_-HSL for 18 h, in comparison to control. Indeed, 95 extracellular proteins were significantly affected by 3O-C_12_-HSL, out of which 41 were upregulated. We further focused on bioinformatics analysis of 33 upregulated proteins triggered by 3O-C_12_-HSL (Table 1, Supplementary Figure S3), while proteins annotated as uncharacterized were not considered. Based on the Search Tool for the Retrieval of Interacting Genes and Proteins (STRING) and NCBI GO analyses in *P. aeruginosa*, the upregulated extracellular proteins could be allocated to functionally distinct groups, including toxic and degradative secreted enzymes; protein and DNA secretion system; synthesis of QS signals; flagellum-dependent cell motility and chemotaxis; adhesion; cell division and cell envelope synthesis; RNA and DNA processes; intracellular metabolism including cellular protein modification, folding processes, and iron homeostasis; virulence; and biofilm formation. Among these 33 upregulated proteins, at least 21 have been previously found to be QS-regulated, where most notable were elastase B, protein hcp1, chitin-binding protein CpbD, 2-heptyl-4(1H)-quinolone synthase, aconitate hydratase B, glutarate-semialdehyde dehydrogenase, two aminopeptidases (lap and pepN), and two flagellar hook-associated proteins (flgL and flgK). In parallel, we identified an array of proteins having toxic, degradative, and enzymatic activity and being also involved in virulence and biofilm formation. In addition, typical adhesins, e.g., the hemagglutinin domain-containing protein cluster, were significantly upregulated in 3O-C_12_-HSL-treated *P. aeruginosa* PA14 (Table 1, Supplementary Figure S3).

**TABLE 1:** Identification of extracellular proteins with high expression levels in *Pseudomonas aeruginosa* PA14 after 18-h treatment with 50 µM 3O-C_12_-HSL compared to the diluent control.

Identified protein	Gene UniProt accession number	Protein UniProt accession number	MW kDa	Fold change	*p*-value	Functional groups of protein^a^	Subcellular location	QS control^b^
Elastase	*lasB*	P14756	54	1.5	<0.00010	X X X	Secreted	Yes
Protein hcp1	*hcp1*	Q9I747	17	1.6	0.0037	XX	Secreted	Yes
Glutarate-semialdehyde dehydrogenase	*davD*	Q9I6M5	52	1.5	0.0043	X	Cytosol	Yes
Flagellar hook-associated protein type 3 FlgL	*flgL*	Q9I4P2	47	1.2	0.0059	X X X X	Secreted	Yes
Extracellular DNA degradation protein EddB	*eddB*	Q9HXA4	83	1.8	0.011	X X X	Secreted	Yes
Aconitate hydratase B	*acnB*	Q9I2V5	94	1.3	0.017	X X	Cytosol	Yes
2-Heptyl-4(1H)-quinolone synthase subunit PqsB	*pqsB*	Q9I4X2	31	2.2	0.023	X X X	Cytoplasm	Yes
Flagellar hook-associated protein 1	*flgK*	Q9I4P3	72	1.1	0.037	X XX X	Secreted flagellum	Yes
Aminopeptidase	*lap*	Q9HZQ8	58	1.2	0.045	X X	Secreted	Yes
Aminopeptidase N	*pepN*	Q9HZC5	100	1.2	0.047	X X X	Secreted	Yes
Haemagg_act domain-containing protein	*PA4625*	Q9HVG6	220	1.5	<0.00010	X X X	Secreted	
Cluster of Haemagg_act domain-containing protein	*PA2462 PA0041*	Q9I120 Q9I791	573,362	1	0.0012	X X X	Secreted cytoplasm	
Probable acyl-CoA thiolase	*PA3925*	G3XD40	41	1.8	0.01	X	Cytosol	
Chaperone protein DnaK	*dnaK*	Q9HV43	68	1	0.035	X X X	Cytosol	
Phage tail assembly protein	*PA0624*	G3XCX2 P003085178.1	13	1.8	0.041	X X		
Chitin-binding protein CbpD	*cbpD*	Q9I589	42	1.4	<0.00010	X X X	Secreted	Yes
Cell division coordinator CpoB	*cpoB*	G3XDB2	29	2	0.01	X X	Periplasm	Yes
Peptidase M48 domain-containing protein	*PA0277*	Q9I6L3	27	3.7	0.013	X X	Secreted	Yes
MaoC-like domain-containing protein	*PA4015*	Q9HX12	17	2	0.032	X	Cytosol	Yes
Methyl-accepting chemotaxis protein CtpL	*ctpL*	Q9HUW6	71	2.1	0.038	X X	Membrane	Yes
Oxygen-dependent coproporphyrinogen-III oxidase	*hemF*	P43898	35	2.4	0.043	X X	Cytoplasm	Yes
30S ribosomal protein S1	*rpsA*	Q9HZ71	62	2	0.011	X	Cytosol	
UPF0339 protein PA0329	*PA0329*	Q9I6G2	12	1.7	0.015	X		
50S ribosomal protein L11	*rplK*	Q9HWC5	15	1.6	0.021	X	Cytosol	
Phosphoethanolamine lipid A transferase	*PA1972*	Q9I2D3 WP003113468.1	62	2.6	0.025	X	Membrane	
Lysyl endopeptidase	*prpL*	Q9HWK6	48	1.2	<0.00010	X X	Secreted	Yes
Serralysin	*aprA*	Q03023	50	1.2	0.018	X X	Secreted	Yes
Peptide methionine sulfoxide reductase MsrA	*msrA*	Q9HUF1	24	4	0.023	X	Cytoplasm	Yes
Peptidase C39 domain-containing protein	*PA1762*	Q9I2X7	29	3.5	0.044	X X X	Membrane	Yes
Rick 17 kDa anti domain-containing protein	*PA3819*	Q9HXI3	19	1.4	0.038	X	Outer membrane	
10 kDa chaperonin	*groS*	P30720	10	1.2	0.039	X	Cytoplasm	
Immunomodulating metalloprotease	*impA*	Q9I5W4	100	1.1	0.0026	X X	Secreted	Yes
PepSY domain-containing protein	*PA2659*	Q9I0H9	11	1.1	0.0095	X X	Secreted	

Functional groups of proteins:

X toxic and degradative secreted enzymes (e.g., proteases and nucleases).

X protein and DNA secretion system.

X synthesis of QS signals.

X flagellum-dependent cell motility and chemotaxis.

X adhesion.

X cell division and cell envelope synthesis.

X RNA and DNA processes (e.g., mRNA and rRNA binding and translation).

X intracellular metabolism including cellular protein modification and folding processes and iron homeostasis.

X virulence.

X biofilm formation.

^a^ Functional groups of proteins.

^b^ QS-controlled product according to earlier transcriptomic, proteomic, and metabolomic studies (Arevalo-Ferro et al., 2003; Nouwens et al., 2003; Schuster et al., 2003; Bains et al., 2012; Chugani et al., 2012; Hare et al., 2012; Scott et al., 2013; Davenport et al., 2015; Mould et al., 2020).

Furthermore, we chose these 33 characteristic proteins, all being upregulated in response to natural 3O-C_12_-HSL exposure, as references for the characterization of the *P. aeruginosa* PA14 response to the compound C00 (Supplementary Table S1), C01 (Table 2), C03 (Supplementary Table S2), and C60 (Supplementary Table S3). Interestingly, out of all tested compounds, treatment with C01 resulted in the most significant difference in the appearance of the 3O-C_12_-HSL-responsive reference proteins. Most of them, 25 out of 33, i.e., approximately 75%, were decreased in comparison to 3O-C_12_-HSL and not changed or even decreased in comparison to diluent control (Table 2). These were the key QS-regulated proteins, e.g., elastase B, protein hcp1, glutarate-semialdehyde dehydrogenase, extracellular DNA degradation protein eddB, 2-heptyl-4(1H)-quinolone synthase, aconitate hydratase B, two aminopeptidases (lap and pepN), two flagellar hook-associated proteins (flgL and flgK), and chitin-binding protein CpbD (Table 2, Supplementary Figure S4). Changes in the PA14 extracellular proteome triggered by treatment with C00, C03, and C60 were similar to those induced by 3O-C_12_-HSL treatment (Supplementary Tables S1–S3). This corroborates our findings with the phenotypic assays, where we showed that compound C01 achieved a more prominent inhibitory effect on virulence traits, e.g., total protease and elastase activity (Figure 3). In summary, 3O-C_12_-HSL has a profound effect on extracellular proteome involved in the pathogenicity of *P. aeruginosa*. Out of the four AHL compounds, C01 achieved a distinct inhibitory effect on the extracellular proteome, which is in line with our data on QS-controlled virulence traits.

**TABLE 2:** Differentially expressed extracellular proteins in *Pseudomonas aeruginosa* PA14 after treatment with 50 µM 3O-C_12_-HSL or compound C01 for 18 h compared to each other or to the diluent control.

Identified protein	Gene UniProt accession number	C01 compared to 3O-C_12_-HSL		C01 compared to diluent control	
		Fold change	*p*-value	Fold change	*p*-value
Proteins displayed quantitative profile as low in C01 and high in 3O-C_12_-HSL and low in C01 and high in control					
Elastase	*lasB*	0.5	<0.00010	0.7	0.013
Protein hcp1	*hcp1*	0	<0.00010	0	<0.00010
Glutarate-semialdehyde dehydrogenase	*davD*	0.08	<0.00010	0.1	<0.00010
Flagellar hook-associated protein type 3 FlgL	*flgL*	0.1	<0.00010	0.1	<0.00010
Extracellular DNA degradation protein EddB	*eddB*	0	<0.00010	0	0.00024
Aconitate hydratase B	*acnB*	0.4	0.0003	0.6	0.017
2-Heptyl-4(1H)-quinolone synthase subunit PqsB	*pqsB*	0	0.00037	0	0.031
Flagellar hook-associated protein 1	*flgK*	0.1	<0.00010	0.1	<0.00010
Aminopeptidase	*lap*	0.1	<0.00010	0.1	<0.00010
Aminopeptidase N	*pepN*	0.5	0.0013	0.6	0.04
Haemagg_act domain-containing protein	*PA4625*	0.3	<0.00010	0.4	<0.00010
Cluster of Haemagg_act domain-containing protein	*PA2462 PA0041*	0.5	<0.00010	0.5	<0.00010
Probable acyl-CoA thiolase	*PA3925*	0.06	<0.00010	0.1	0.0027
Chaperone protein DnaK	*dnaK*	0.8	0.00028	0.8	0.01
Phage tail assembly protein	*PA0624*	0.09	0.001	0.2	0.03
Proteins displayed quantitative profile as low in C01 and high in 3O-C_12_-HSL and no difference between C01 and control					
Chitin-binding protein CbpD	*cbpD*	0.7	<0.00010	1	0.5
Cell division coordinator CpoB	*cpoB*	0.5	0.027	1.7	0.092
Peptidase M48 domain-containing protein	*PA0277*	0	0.0052	0	0.35
MaoC-like domain-containing protein	*PA4015*	0.3	0.013	0.6	0.32
Methyl-accepting chemotaxis protein CtpL	*ctpL*	0.2	0.034	0.6	0.4
Oxygen-dependent coproporphyrinogen-III oxidase	*hemF*	0.2	0.034	0	0.088
30S ribosomal protein S1	*rpsA*	0.3	0.0074	0.5	0.18
UPF0339 protein PA0329	*PA0329*	0.4	0.01	0.7	0.31
50S ribosomal protein L11	*rplK*	0.6	0.054	1	0.52
Phosphoethanolamine lipid A transferase	*PA1972*	0	0.0018	0	0.088
Proteins displayed the same quantitative profile in 3O-C_12_-HSL and C01 and as low in control					
Lysyl endopeptidase	*prpL*	1	0.5	1.3	0.00014
Serralysin	*aprA*	1.2	0.17	1.5	0.00058
Peptide methionine sulfoxide reductase MsrA	*msrA*	0.6	0.27	2.3	0.24
Peptidase C39 domain-containing protein	*PA1762*	0.4	0.21	1.6	0.46
Rick 17 kDa anti domain-containing protein	*PA3819*	0.9	0.3	1.3	0.15
10 kDa chaperonin	*groS*	0.9	0.18	1.1	0.35
Proteins displayed quantitative profile as high in C01 and low in 3O-C_12_-HSL and low in control					
Immunomodulating metalloprotease	*impA*	1.2	0.015	1.3	<0.00010
PepSY domain-containing protein	*PA2659*	1.5	<0.00010	1.7	<0.00010

### Molecular modeling

**FIGURE 4:**
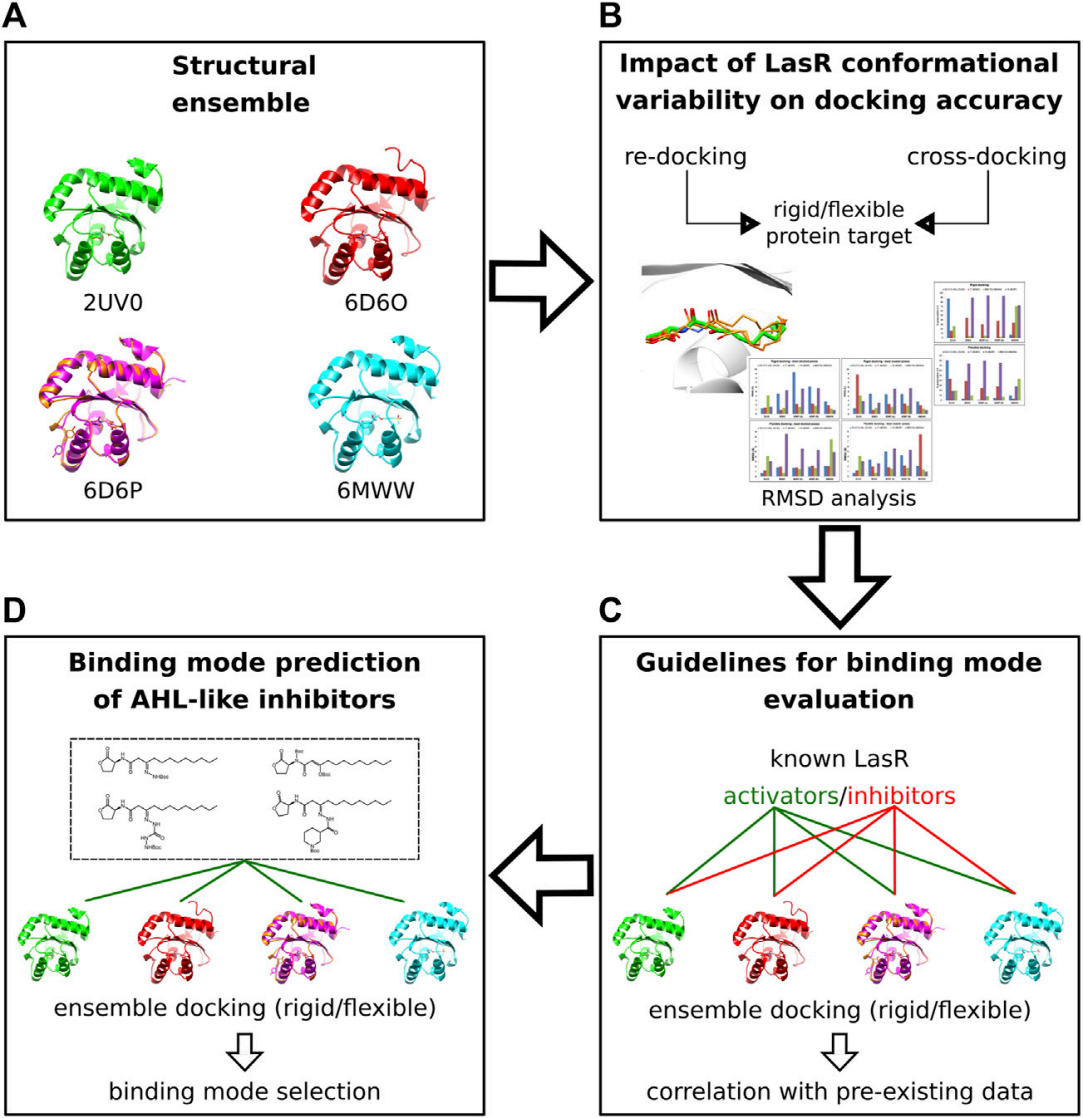
Molecular docking workflow. **(A)** Five crystal structures of LasR in complex with different agonists were selected. **(B)** The selected ensemble of LasR agonist complexes was used to assess re-docking and cross-docking protocols either using rigid protein structures or side chains of Leu36, Tyr47, Ile52, and Arg61 flexible. **(C)** Rigid and flexible docking simulations of known LasR modulators were performed into the selected ensemble of protein structures to determine guidelines for binding mode evaluation. **(D)** Docking and binding mode analysis of the new AHL compounds were performed using the assessed docking protocols and the criteria established for binding mode evaluation.

By using either pre-existing structural or biochemical data, we performed docking validation, measured the impact of protein flexibility on the docking precision, and determined the criteria to evaluate and select docking poses of the new AHL inhibitors (see Discussion). The docking workflow is summarized in Figure 4.

### Accounting for LasR flexibility and L3 loop deviations

To include protein flexibility, we selected four LasR agonist complexes (PDB ID: 2UV0, 6D6O, 6D6P, and 6MWW), representing key LasR-LBD conformational states that were detected either with potent or weak agonists (Paczkowski et al., 2019; O'Reilly et al., 2018; Bottomley et al., 2007). A total of five target structures were employed to perform either rigid or flexible re-docking and cross-docking simulations (Supplementary Table S4, Materials and methods). In flexible docking, side chains of Leu36, Tyr42, Ile52, and Arg61 were allowed to rotate.

### Docking assessment

Pre-existing structural and biochemical data were used to assess different docking protocols for accurate binding mode prediction of LasR ligands.

#### Rigid re-docking and cross-docking of co-crystallized LasR agonists

Re-docking and cross-docking experiments were initially evaluated using rigid target structures. Re-docking of LasR agonists in their cognate structures showed a 100% predictive accuracy (Ballante and Marshall, 2016). Importantly, all the top-scored docked poses (best-docked) belonged to the largest cluster of predicted binding modes (i.e., they are also best-cluster poses) and showed an RMSD < 2 Å from the experimental coordinates (Supplementary Figures S5A, B, Supplementary Tables S5, S6). In the case of the LasR autoinducer complex (PDB ID: 2UV0), we performed re-docking either by maintaining or removing a bridging water molecule involved in hydrogen bonding between the 3-oxo group of the ligand’s head and Arg61 (see Materials and methods). As expected, inclusion of the water molecule increased the predictive performance when re-docking 3O-C_12_-HSL into 2UV0 (the RMSD value decreased from 1.29 Å to 0.77 Å, Supplementary Tables S5, S6). The results from rigid re-docking confirmed the reliability of predicting the binding pose of LasR agonists in their cognate structures. Rigid cross-docking (docking of LasR activators into non-native protein structures) was then assessed to measure the impact of using non-cognate protein target conformations on binding mode prediction.

Compared to rigid re-docking, cross-docking’s best-docked or best-cluster poses showed higher RMSD values from the experimental mode of binding (Supplementary Figures S5A, B, Supplementary Tables S5, S6). Analysis of the number of docking poses within 2 Å from the experimental one showed, except for 6D6O, high percentages of similar-to-experiment poses in the case of docking into the native protein structure (Supplementary Figure S6A and Supplementary Table S9). Good percentage values were also determined in the case of docking into target structures endowed with a similar L3 loop conformation. Conversely, lower percentages of similar-to-experiment poses were detected when docking the natural autoinducer (3O-C_12_-HSL) and BB0126 into non-cognate protein structures, as well as compounds 17 and 19, into 2UV0.

The results from rigid re-docking and cross-docking displayed a substantial influence of the used 3D protein structure on the docking performance, indicating that different conformational states of LasR should be used to achieve a better model accuracy and to increase the number of consistent docked poses for prospective analyses.

#### Flexible re-docking and cross-docking of co-crystallized LasR agonists

We evaluated flexible re-docking and cross-docking, by allowing Leu36, Tyr47, Ile52, and Arg61 to rotate, with the aim to determine the impact of limited side chain flexibility on the docking performance. Similar to rigid re-docking, best-docked and best-cluster poses from flexible re-docking showed generally RMSD values <2 Å from the experimental mode of binding (Supplementary Figures S5C, D, Supplementary Tables S7, S8). In the case of 6MWW, the best-cluster pose outperformed the best-docked one. Flexible cross-docking outperformed rigid cross-docking in predicting the best-docked pose of 3O-C_12_-HSL into non-native protein structures (compare Supplementary Figure S5A with Supplementary Figure S5C). Analysis of the number of docking poses within 2 Å from the experimental one showed still good percentages from flexible re-docking. However, compared to rigid cross-docking, flexible cross-docking never failed to produce at least one good pose (compare Supplementary Figure S6A with Supplementary Figure S6B and Supplementary Table S9 with Supplementary Table S10). These results indicated that explicit flexibility of Leu36, Tyr47, Ile52, and Arg61 can also facilitate the prediction of experiment-like binding modes of LasR ligands.

#### Binding mode prediction of known LasR ligands

Taking into account the results obtained in predicting the experimental poses of LasR agonists, we investigated the performance of docking LasR agonists and antagonists which are devoid of structural data. To better evaluate the consistency between the obtained docked poses and the experimental data, we mainly chose ligands that had been tested both in wild-type and in mutant LasRs.

1) AHL-like ligands with modified head structures: the aniline analog (originally reported as compound 1) of 3O-C_12_-HSL (LasR autoinducer) is a LasR inhibitor in WT but a LasR activator in Trp60Phe, Tyr56Phe, and Ser129Ala mutants (Gerdt et al., 2014). The experiments indicate that Tyr56 and Ser129 are responsible to orient and hold compound 1 in a way that its aniline head group clashes or lacks H bonding with Trp60, thus determining LasR inhibition. Rigid docking of compound 1 in 2UV0 could reproduce a binding mode that is consistent with the experimental data, by predicting H bonding between the amide carbonyl and Tyr56/Ser129, as well as no favorable interactions with Trp60 (Supplementary Figure S7A).

Due to its high similarity with 3O-C_12_-HSL, it can be expected that compound 1 also forms H bonds via a water molecule to Arg61. As seen for the endogenous activator, inclusion of bridging water facilitated docking toward coherent docked poses, all of which belonged to a unique cluster (Supplementary Figure S7B). Replacement of the aniline head with 2-aminocyclohexanol (originally reported as compound 4) led to a LasR agonist (Smith et al., 2003), most likely due to the restored ability to hydrogen bond with Trp60 through the hydroxyl group, a key interaction that docking into 2UV0 was able to reproduce (Supplementary Figure S7C).

2) AHL-like antagonists containing bulky tail structures: Compounds 7h and 7o are LasR antagonists endowed with an indol and bromophenyl tail, respectively (Geske et al., 2005). Rigid docking of the two antagonists into 2UV0 was not able to provide binding modes in agreement with LasR antagonism. Indeed, both compounds were consistently modeled to establish key interactions typically seen in LasR agonist complexes, e.g., H bonding between the head groups and Trp60. Conversely, rigid docking into 6D6P predicted binding modes in line with LasR antagonism, e.g., H bonding between the ligands and Tyr56/Ser129 but weak/no H bonding with Trp60 (Supplementary Figure S8).
3) Non-native LasR antagonists: Compounds V-06-018 and 39 are two β-keto amides and potent LasR antagonists (Manson et al., 2020). V-06-018 has a phenyl head group and a nine-carbon tail, whereas 39 has a furan head group and contains a 10-carbon tail. Both compounds were tested in antagonism and agonism assays in three LasR mutants (Tyr56Phe, Trp60Phe, and Ser129Ala) and showed a reduced antagonism in all the three mutants (Manson et al., 2020). Experiments suggested the role of Tyr56 and Ser129 in establishing hydrogen bonds with one of the two linker carbonyls in V-06-018 and 39, as well as poor interactions or steric clashes between the modified head groups and Trp60. Flexible docking of V-06-018 and 39 into 2UV0 predicted a binding mode consistent with the experimental data (Supplementary Figure S9).

### Binding mode prediction of the AHL compounds

Rigid and flexible cross-docking protocols were performed to predict the binding mode of compounds C00, C01, C03, and C60. Like 3O-C_12_-HSL, compounds C00, C03, and C60 possess a homoserine lactone moiety at the head region and a central amide group as a linker. This structural similarity indicates the likelihood of a binding mode and interaction pattern of the linker mimicking those of the autoinducer, i.e., H bonding with Tyr56, Ser129, and Asp73, but such that the homoserine lactone displaces from or collides with Trp60 due to the different tail groups. This criterion was used to evaluate the quality of the docked poses of compounds C00, C03, and C60. Conversely, compound C01 has an imide group as a linker, suggesting that either one of the two carbonyl oxygens from the linker or one from the other carbonyl groups in the molecule could be involved in H-bond interactions with Ser129 and/or Tyr56. Accordingly, docked poses of C01 showing a hydrogen bond between one of its carbonyl oxygens, Ser129 and/or Tyr56, were prioritized during visual inspection.

**FIGURE 5:**
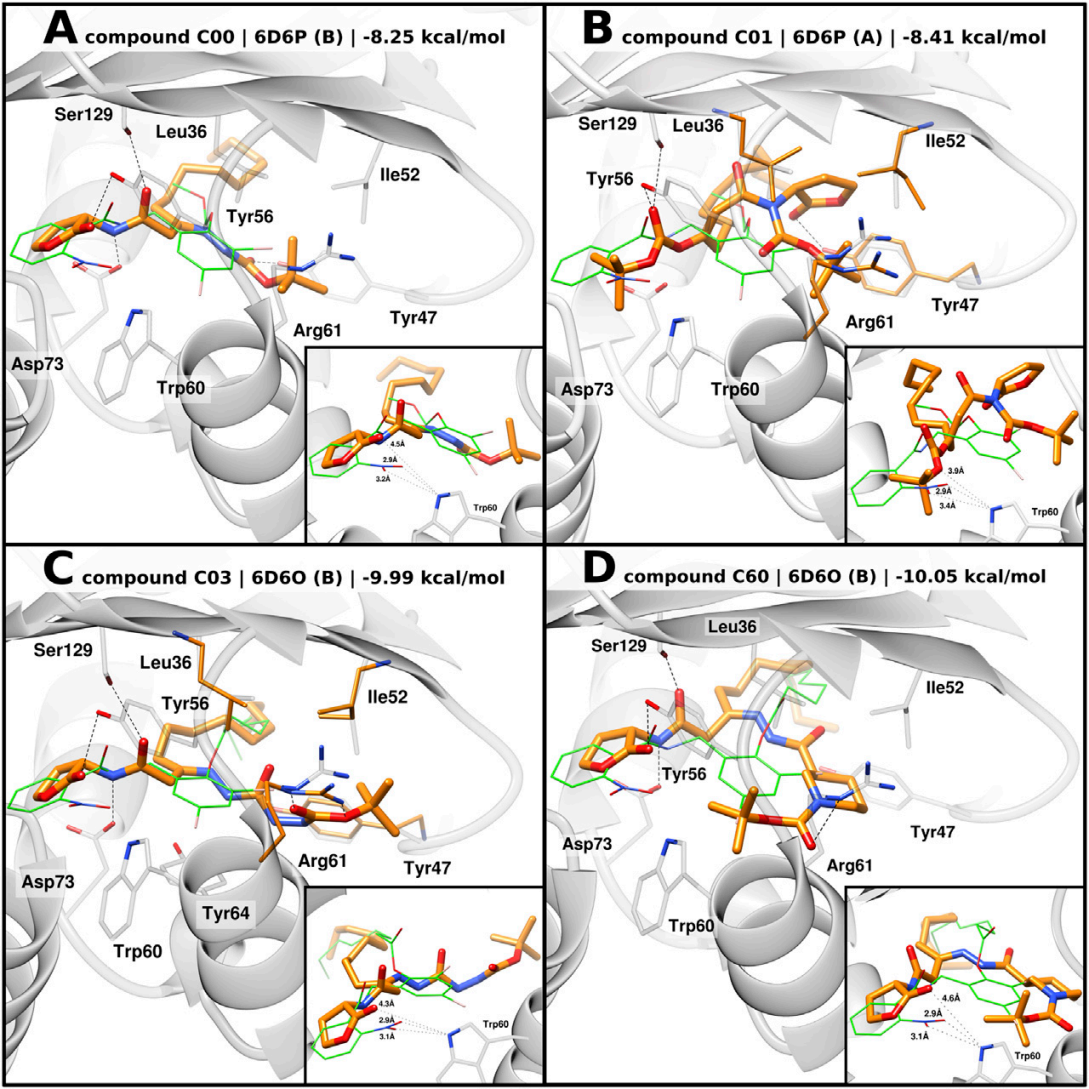
Predicted binding mode of AHL compounds in the ligand-binding pocket of LasR. AHL compounds are shown as orange sticks. Selected protein structures and the native co-crystallized ligand are shown as gray representations and green wires, respectively. Selected side chains are shown as gray sticks; in the case of flexible docking, modeled side chains of Leu36, Tyr47, Ile52, and Arg61 are shown as orange sticks. Putative hydrogen bonds are shown as black dashed lines. Smaller boxes: close-up of predicted distances, shown as black dotted lines, between AHL compounds and Trp60. Experimental distances between the native co-crystallized ligands (green wires) and Trp60 are shown as a reference. **(A), (B)** Docking poses of compounds C00 and C01 into 6D6P, respectively, superimposed with the original co-crystallized ligand compound 19; **(C), (D)** docking poses of compounds C03 and C60 into 6D6O, respectively, superimposed with original co-crystallized ligand compound 17.

Docking of C00 into 6D6P best reproduced the hydrogen bonding network between the amide linkers, Ser129 and Asp73, as well as the homoserine lactone head being displaced from Trp60 (Figure 5A). It can be speculated that the head group is shifted due to the t-butoxycarbonyl hydrazine moiety which, in 6D6P, can be housed between Tyr47, Ile52, Leu36, Tyr64, and Arg61, driving a pivoting motion at the linker region. Compared to 2UV0, 6D6P shows that Arg61 flipped out from the ligand-binding domain and the L3 loop in the “out” conformation, thus allowing larger moieties to accommodate into the pocket. Tyr47 from the L3 loop is predicted to establish a H bond with the tert-butyloxycarbonyl group. Docking poses of compounds C03 and C60 into 6D6O show H-bond interactions between their amide linkers, Ser129 and Asp73, and their head groups shifted away from Trp60 (Figures 5C, D). The binding modes of C03 and C60 into 6D6O suggest that their t-butoxycarbonyl hydrazine carbohydrazide and t-butoxycarbonyl nipecotic acid hydrazine moieties can accommodate between Tyr47, Ile52, Leu36, Tyr64, and Arg61. Like 6D6P, 6D6O shows that Arg61 flipped out from the ligand-binding domain and the L3 loop in the “out” conformation. In case of compound C03, the selected docking pose was generated from flexible docking, showing Arg61 engaged in H-bond interactions with the inhibitor. Likewise, docking of C60 also predicted H-bond interaction with Arg61.

Similar to 3O-C_12_-HSL, docking of C00, C03, and C60 modeled their acyl tail chains into the hydrophobic pocket.

In the case of compound C01, flexible docking of C01 into 6D6P was able to produce a coherent binding mode. Conversely to the other compounds, the carbonate ester moiety of C01 is predicted to act as the head group with the carbonyl oxygen being able to establish H-bond interactions with Ser129 and/or Tyr56 (Figure 5B). The tert-butyloxy moiety is modeled to point toward Trp60 suggesting weak H-bond interaction with Trp60 and a possible shielding effect of the tert-butyl group. The homoserine lactone group of C01 is modeled to engage H bonding with Tyr47 from the L3 loop “out” and the acyl tail enclosed in the hydrophobic pocket.

## Discussion

**FIGURE 6:**
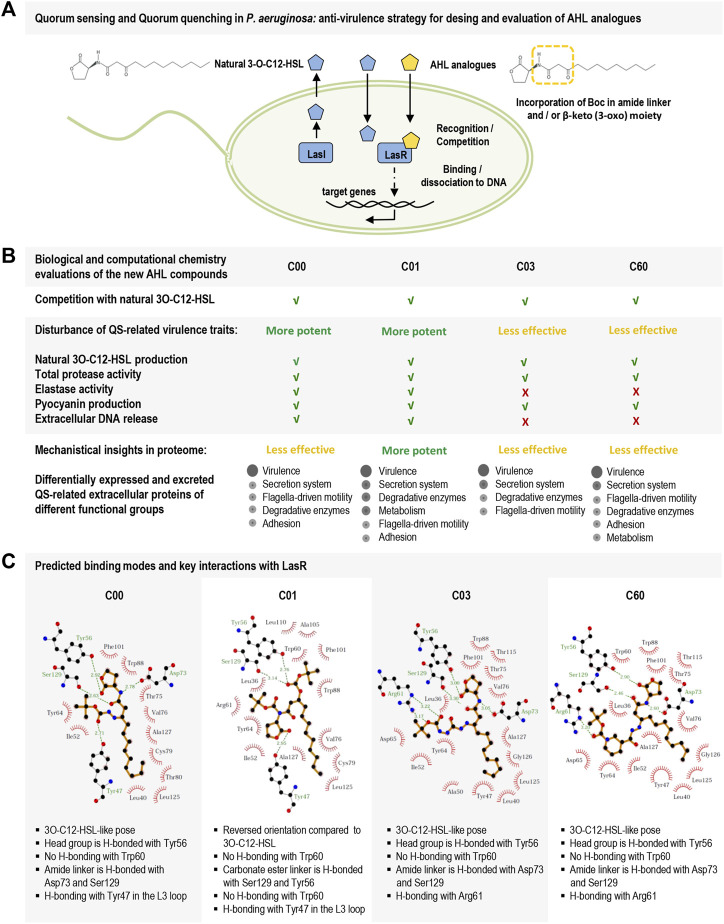
Schematic overview of the study on new AHL analogs with characteristics of QSIs in *Pseudomonas aeruginosa.* **(A)** Hierarchal top circuit in the QS system of *Pseudomonas aeruginosa*, LasI/LasR, and its signal molecule 3O-C_12_-HSL (blue pentagon) that together with other circuits in the QS system provides cell–cell communication between bacteria and control pathogenic lifestyle, host–pathogen interactions, and development of antibiotic resistance. The scheme presents our strategy for the design of AHL analogs (yellow pentagon) with the incorporation of a Boc group in amide linker and β-keto (3-oxo) moiety. **(B)** Biological evaluations of the new AHL analogs, i.e., compounds C00, C01, C03, and C60. C01 displayed the most potent LasR-based antivirulence activity against the pathogenic bacteria *Pseudomonas aeruginosa*. **(C)** LigPlot + interaction diagrams and predicted key interactions based on docking results of the new AHL analogs into the ligand-binding domain of LasR.

In this study, we report on the design and testing of new *P. aeruginosa* AHL analogs (C00, C01, C03, and C60) for their ability to act as QSIs. Particularly, compound C01 decreased LasR-dependent activity and competed efficiently with 3O-C_12_-HSL, thereby significantly reducing the production of, for instance, the cognate autoinducer, virulence traits (like protease and elastase activity, pyocyanin production, and extracellular DNA release), and the expression of QS-regulated extracellular proteins. We combined experimental data with computational chemistry to assess the advantage of using a multi-conformational docking procedure for binding mode prediction of LasR modulators and set criteria for docking pose evaluations. A multi-conformational docking protocol was then applied to predict the binding mode of the hit compounds (C00, C01, C03, and C60) and to delineate key interactions with LasR (Figure 6).

QS is a cell-to-cell communication used by many clinically relevant bacteria to regulate processes associated with the pathogenesis and development of antibiotic resistance. QS signaling allows bacteria to control their virulent behavior and coordinate the attack on a competing microorganism or the host when the bacterial population is adequately high. This strategy ensures the survival of the pathogens and the escape from host defenses and drug therapy. QS inhibition has, therefore, been considered an attractive new strategy to combat bacterial infections and is commonly referred to as quorum quenching. Many Gram-negative pathogens, including *P. aeruginosa*, rely on QS communication via Las and Rhl circuits, which use AHL as signals, or autoinducers. This gives a prospect of small-molecule intervention of the AHL-based QS system, (Galloway et al., 2012; Liao et al., 2022), where much emphasis has been placed on the discovery and development of the LasR receptor inhibitors, since the Las system is generally considered to be situated at the top of the QS hierarchy in *P. aeruginosa* (Kalia, 2013). A toolbox of QSI compounds has been prepared by chemical synthesis of non-native AHL derivates and mimics (Geske et al., 2008; Manson et al., 2020; Hodgkinson et al., 2012; O'Loughlin et al., 2013), designed by chemical strategies (Amara et al., 2009), identified by computer-aided drug design and *in silico* approaches (Muh et al., 2006; Yang et al., 2009; Vetrivel et al., 2021; Magalhaes et al., 2022), obtained from natural sources (Paczkowski et al., 2017; Hernando-Amado et al., 2020; Shukla et al., 2021), and by screening of databases for small-molecule libraries (Tan et al., 2013).

Incidentally, in recent years, a range of QSIs were designed based on the AHL analogs with modifications positioned in the head group, amide linker, β-keto (3-oxo) moiety, or tail group (Figure 1A). In the naturally amphiphilic AHL, the head group defines ligand-binding orientation in the LasR pocket, while the tail group contributes to ligand association within the LasR pocket. Depending on its length, the acyl chain occupies the binding pocket in different configurations: a short chain is extended and points toward the L3 loop, whereas a long chain is bent and faces the interior of the pocket. The amide linker provides a strongly polar characteristic to AHL and a high potential to serve as a H-bound donor or acceptor (Li and Nair, 2012). Since AHL analogs are structurally alike the native ligands, it can be anticipated that they possess a similar affinity to LasR. Still, based only on the structure, it is difficult to predict whether they will display agonistic or antagonistic activity. However, approaches for molecular modeling, competition, and phenotypic assays may bring more information on whether they will show characteristics of QSIs.

Here, we designed four new AHL analogs in which either amide linker or β-keto (3-oxo) moiety or both were modified by the incorporation of a tert-butoxycarbonyl group (Boc), while the head and tail were retained intact (Figure 1). Several compounds with anti-QS activity have been discovered, when the amide linker (Frezza et al., 2008; Boukraa et al., 2011; Stevens et al., 2011) or the β-keto (3-oxo) moiety (Forschner-Dancause et al., 2016) had been modified. It has also been shown that Boc modification of formyl peptide leukocytes (Johansson et al., 1993; Stenfeldt et al., 2007) and Wnt5a-derived peptide resulted in the discovery of antagonists that appeared to block the cell activity more than receptor binding (Jenei et al., 2009; Yadav et al., 2021). Thus, we suggest here that the central connective parts of 3O-C_12_-HSL can be incorporated with Boc, and this may result in LasR modulators with anti-QS activity.

We, thus, studied the ability of AHL compounds and natural 3O-C_12_-HSL to compete with each other when binding to LasR using the *P. aeruginosa*-derived biosensor PA14-R3. Interestingly, AHL compounds were found to be able to compete with 3O-C_12_-HSL for perception by LasR as they limited the activity of LasR in the biosensor, both alone and in competition with 3O-C_12_-HSL.

Next, we investigated the effect of AHL compounds on the production of 3O-C_12_-HSL *in vivo* in wild-type *P. aeruginosa*. Our data demonstrate that all four AHL compounds caused lower production of 3O-C_12_-HSL in bacteria and that compound C01 appeared more potent than the other three analogs.

Furthermore, using phenotypic assays, we tested the effect of our compounds on the activity and production of various virulence traits, such as proteases, elastase, pyocyanin, and extracellular DNA in *P. aeruginosa* PA14. Many extracellular enzymes with proteolytic activity are regulated by Las and Rhl circuits, including the 33-kDa elastase B being involved in the pathogenesis by acting on many substrates, e.g., elastin, collagen types II and IV, fibronectin, and immunoglobulin A, thereby degrading mucosal and connective tissues and preventing bacterial clearance (Pont et al., 2022; Tummler, 2022). *Pseudomonas aeruginosa* also secretes pyocyanin, which is a blue–green-colored phenazine, redox-active, toxic secondary metabolite. It is regulated by the QS system and helps *P. aeruginosa* compete with other microbes and mammalian cells. It also facilitates the release of extracellular DNA, a key nutrient source, an essential component for the exchange of genetic information, and a biofilm component that contributes to its stability and cohesion (Das and Manefield, 2012). The outcome of our phenotypic studies is intriguing, as all four AHL compounds did reduce the total activity of proteases and pyocyanin production. Moreover, compounds C00 and C01, but not C03 and C60, decreased the elastase activity and the amount of released extracellular DNA. This demonstrates the potential of our AHL compounds as a QSI and an antivirulence modulator of *P. aeruginosa*.

This encouraged us to explore further the molecular mechanisms and delineate alterations in the *P. aeruginosa* PA14 extracellular proteome in response to AHL compounds using MS-based quantitative proteome profiling. We indeed identified an ensemble of differentially expressed extracellular proteins in PA14 as a response to AHL compounds in comparison to 3O-C_12_-HSL. First, natural 3O-C_12_-HSL had a profound effect on the extracellular proteome involved in the pathogenicity of bacteria, as approximately 9.5% of extracellular proteins were significantly affected by 3O-C_12_-HSL, either upregulated or downregulated. To our surprise, the most potent compound was again C01, which corroborates our findings with the phenotypic assays. Thus, the treatment of *P. aeruginosa* with compound C01 resulted in the most significant alteration in the appearance of the 3O-C_12_-HSL-responsive reference proteins; up to 75% of these proteins were decreased in comparison to 3O-C_12_-HSL, or unchanged, or even lowered as compared with diluent control. They are crucial in virulence, secretion, toxicity, enzymatic tissue degradation, flagellum-dependent motility, adhesion, metabolism, synthesis of QS signals, and biofilm formation. Among these proteins were elastase B, protein hcp1, glutarate-semialdehyde dehydrogenase, extracellular DNA degradation protein eddB, 2-heptyl-4(1H)-quinolone synthase, aconitate hydratase B, two aminopeptidases (lap and pepN), two flagellar hook-associated proteins (flgL and flgK), and chitin-binding protein CpbD.

*Pseudomonas aeruginosa* is equipped with a very potent arsenal of virulence determinants and systems that allow the pathogen to adapt in a host niche, escape host defense, and develop infectious diseases. Among them, the QS communication system and types 1, 2, 3, and 6 secretion systems (T2SS, T3SS, and T6SS) for transport of exceptionally large proteins are of importance. Protein hcp1 is the hemolysin co-regulated protein 1 and the effector of T6SS that translocates at least seven antibacterial toxins Tse1–Tse7 directly inside host cells or prokaryotic competitors providing fitness and survival advantages to the bacteria. T2SS secretes exotoxin A, chitin-binding protein CpbD, and proteases in the extracellular milieu, including elastases A and B, phospholipase C, and protease IV. Like T6SS, T3SS translocates effector proteins directly into host cells, e.g., the Exo family toxins that inactivate small GTPases, which leads to disruption of the cytoskeleton and induction of apoptosis. The relationship between QS network elements and certain types of secretion systems has been described in *P. aeruginosa* and other pathogens (Pena et al., 2019; Sauvage and Hardouin, 2020).

The proteolytic extracellular enzymes, elastase A, elastase B, alkaline protease, and aminopeptidases, are well known as leading virulence products supporting bacteria to enter into and survive in the host by avoiding the immune host system (Tummler, 2022). Elastase B cooperates with alkaline protease to degrade exogenous flagellin and prevent flagellin-mediated immune recognition (Casilag et al., 2016). *Pseudomonas* spp. possess polar flagella that provide chemotaxis-driven motility, an important fitness for bacterial colonization and virulence. The long flagellin filament is attached to the hook–basal body complex by two junctional flagellar hook-associated proteins, flgL and flgK. Regulation of flagella function likely depends on cell density, but the link between QS and flagella function is still unclear in *P. aeruginosa.* T6SS may be an alternative mode to sense cell density and then regulate flagellar motility in *Pseudomonas* species (Bouteiller et al., 2021). Among differentially expressed extracellular QS-related proteins of *P. aeruginosa* as a response to AHL analogs, we identified such playing roles in nutrient acquisition and metabolism. Indeed, QS leads to a global metabolic readjustment in *P. aeruginosa* (Davenport et al., 2015).

Out of four AHL compounds, C01 achieved the most prominent inhibitory effect on *P. aeruginosa* based on the competitive, phenotypic assays and quantitative extracellular proteome profiling. This AHL analog has two Boc incorporations located at the amide linker and in β-keto (3-oxo) moiety, which may have played a key role in the anti-QS activity. In compounds C00, C03, and C60, employing the amide linker in different ways evidently makes them less efficient as QSIs (Figures 1, 6). It has been reported previously that the substitution of the amide bond by bioisosteric compounds, e.g., sulfonamides, ureas, and sulfonylureas, generated a new family of active compounds capable of inhibiting QS-regulated responses in the fish pathogen *Vibrio fisheri* (Stevens et al., 2011). AHL mimics at aryl β-keto esters may also act as antagonists by competing natural AHL and the inhibitor of QS-controlled bioluminescence in another marine pathogen, *V. harveyi* (Forschner-Dancause et al., 2016). A future aim would be to expand the panel of Boc-modified AHL analogs and perform structure–activity relationship studies to aid in our knowledge of rational drug design and for the development of a novel QSI.

Due to the lack of structural data for LasR proteins bound to inhibitors, the application of structure-based computational techniques for the discovery of new antagonists is challenging. However, pre-existing structural and biochemical data can be used to assess and optimize the docking performance and guide the criteria for assessing the quality of the docked poses (visual inspection). In brief, a) crystallography data show that LasR activators establish, through their head and linker groups, H bonding with Tyr56, Trp60, Ser129, and Asp73 (Bottomley et al., 2007; O'Reilly et al., 2018; Paczkowski et al., 2019). Importantly, modifications to the head portion of an agonist, which disrupt H bonding or determine steric clashes with Trp60, may reduce potency or switch the activity to antagonism. As for example, small structural modifications to the head group of 3O-C_12_-D12, a LasR agonist, led to partial agonists and antagonists (Smith et al., 2003; Moore et al., 2015). Similarly, switching the ligand’s head from lactone to benzene or from lactone to thiolactone, with maintaining the same tail, flips the activity (Gerdt et al., 2014).

b) Site-directed mutagenesis has revealed the main role of Tyr56, Trp60, and Ser129 in modulating the behavior of a LasR ligand (Gerdt et al., 2014), indicating that Tyr56 and Ser129 drive ligand orientation and affect the interaction between the ligand head and Trp60, which mainly governs the ligand activity. Engagement of Tyr56, Trp60, and Ser129 in the antagonism was further confirmed by LasR mutant antagonist and agonist experiments of non-native LasR antagonists, e.g., compounds V-O6-018 and 39 (Manson et al., 2020).
c) The LasR binding pocket is endowed with significant flexibility, and ligands can induce protein conformational changes that affect protein stability and ligand activity. For example, two distinct conformers (“in” and “out” conformations) of the L3 loop were experimentally determined and correlated to the ligand size and potency. The results from the structural, activity, and thermal assays indicated that deviations of the L3 loop from the “in” conformation, characterizing the LasR autoinducer complex, generally correlate with lower protein stability and ligand potency (O'Reilly et al., 2018). Despite the unavailability of crystallographic data for LasR inhibitors, it is reasonable to assume that small-molecule antagonists can also induce such destabilizing protein conformational changes.
d) Combined mutagenesis studies with chemical synthesis and crystallography revealed that Thr75, Tyr93, and Ala127 stabilize non-canonical binding modes of weak agonists, where the head group is flipped compared to that of the native autoinducer (Paczkowski et al., 2019).

Based on the experimental evidence, it is reasonable to expect that a ligand can antagonize LasR either by poorly interacting with Tyr60 or by inducing the ligand-binding domain (LBD) to destabilize conformational changes or both. Accordingly, accounting for protein flexibility when docking small molecules into LasR would improve the representation of induced fit as well as the docking accuracy in predicting the binding mode of agonists and antagonists. Moreover, Asp73, Tyr56, and Ser129 have generally shown a key role in the binding of LasR modulators, as they act as anchor points through which a ligand undergoes a pivoting motion that orients itself in the binding pocket and consequently determines the interaction between the head group and Trp60. Thus, favorable hydrogen bonding between the compound’s linker region and Asp73, Tyr56, or Ser129 should be carefully considered during the visual inspection of docking poses of LasR modulators. We used such pre-existing knowledge of known LasR ligands as guidelines to assess molecular docking and determine the criteria to evaluate the quality of predicted binding modes. Docking assessment using co-crystallized LasR agonist complexes and pre-existing biochemical data of modulators indicated that a multi-conformational docking approach is necessary to achieve satisfactory docking accuracy. Moreover, prioritization of docking poses showing H bonding between the linker region and Asp73, Tyr56, or Ser129 facilitated the detection of binding modes of extensively studied LasR agonists and antagonists, which are in agreement with the experimental results. Accordingly, we performed rigid and flexible cross-docking protocols to predict the binding mode of compounds C00, C01, C03, and C60 and prioritized docking poses showing H bonding with Tyr56, Ser129, or Asp73, but no attractive interaction with Trp60. Such a strategy allowed finding binding modes that were consistent with the current knowledge on LasR modulator recognition and useful to further clarify key molecular interactions in LasR–antagonist complexes.

In conclusion, we report here on the design and function of structurally novel AHL analogs incorporating a Boc group in amide and β-keto (3-oxo) moiety. Biological and computational chemistry evaluation of these compounds led to the identification of one of these compounds, C01, displaying the most potent LasR-based antivirulence activity against pathogenic bacteria *P. aeruginosa* (Figure 6). Overall, our findings point out a new group of non-natural small molecules that could be relevant to the development of QSIs against the highly virulent superbug *P. aeruginosa.* The data obtained from our studies may provide a new avenue for manipulation of QS-related responses in human opportunistic pathogen *P. aeruginosa* and open the door for rational design of next-generation small-molecule tools, structure–activity relationship studies, and discovery of an efficient QSI.

## Data Availability

The mass spectrometry data has been deposited to the ProteomeXchange Consortium (www.proteomexchange.org) via the PRIDE partner repository with the dataset identifier PXD045881.

## Abbreviations

3O-C_12_-HSL, *N*-3-oxo-dodecanoyl-L-homoserine lactone; C_4_-HSL, *N*-butyryl-L-homoserine lactone; AHL, *N*-acylhomoserine lactone; A280, A495, A600, and A690, absorbances at 280, 495, 600, and 690 nm wavelength, respectively; dsDNA, double-stranded DNA; DTT, dithiothreitol; IAA, iodoacetamide; LB, Luria-Bertani medium; LBD, ligand-binding domain; LC–MS/MS, liquid chromatography tandem mass spectrometry; MINS, minimal medium; MOPS, 3-morpholinopropane sulfonic acid; RMSD, root-mean-squared deviation; RT, room temperature; TEM, transmission electron microscopy; QS, quorum sensing; QSIs, quorum-sensing inhibitors.
