# Supplementary material for: Modified N-acyl-L-homoserine lactone compounds abrogate Las-dependent quorum-sensing response in human pathogen Pseudomonas aeruginosa
Source: Front Mol Biosci. 2023 Oct 16;10:1264773. doi: 10.3389/fmolb.2023.1264773 (PMC10613653; doi:10.3389/fmolb.2023.1264773)
Supplement: Supplementary file 1 [file DataSheet1.docx]

***Supplementary Material***

**Modified** ***N*-acyl-L-homoserine lactone compounds abrogate Las-dependent quorum sensing response in human pathogen *Pseudomonas aeruginosa***

**Flavio Ballante ^1^, Maria V. Turkina ^2^, Maria Ntzouni ^3^, Karl-Eric Magnusson ^2^,**

**Elena Vikström ^2^ ***

*^1^ Chemical Biology Consortium Sweden (CBCS), Science for Life Laboratory, Department of Medical Biochemistry and Biophysics, Karolinska Institutet, Stockholm, Sweden,*

***^2^*** *Department of Biomedical and Clinical Sciences, Faculty of Medicine and Health Sciences, Linköping University, Sweden,*

***^4^*** *Core Facility, Faculty of Medicine and Health Sciences, Linköping University, Sweden*

* Correspondence:

Elena Vikström

Linköping University

Faculty of Medicine and Health Sciences

Department of Biomedical and Clinical Sciences

Linköping SE-581 85 Sweden

[elena.vikstrom@liu.se](mailto:elena.vikstrom@liu.se)

Total number of supplementary figures, tables and text: 21

(Including 1 text with methods , 9 figures, 10 tables, and 1 text with references)

**METHODS**

**Molecular Docking.** Ligands were protonated at physiological pH by means of the *majormicrospecies* function from the cxcalc tool (version 22.12.0-1538, ChemAxon, <https://www.chemaxon.com>). Result molecules were visually inspected and the lowest energy conformers were calculated through the cxcalc’s *leconformer* function using the hyperfine option and optimization limit set to 1.

**FIGURES**

**
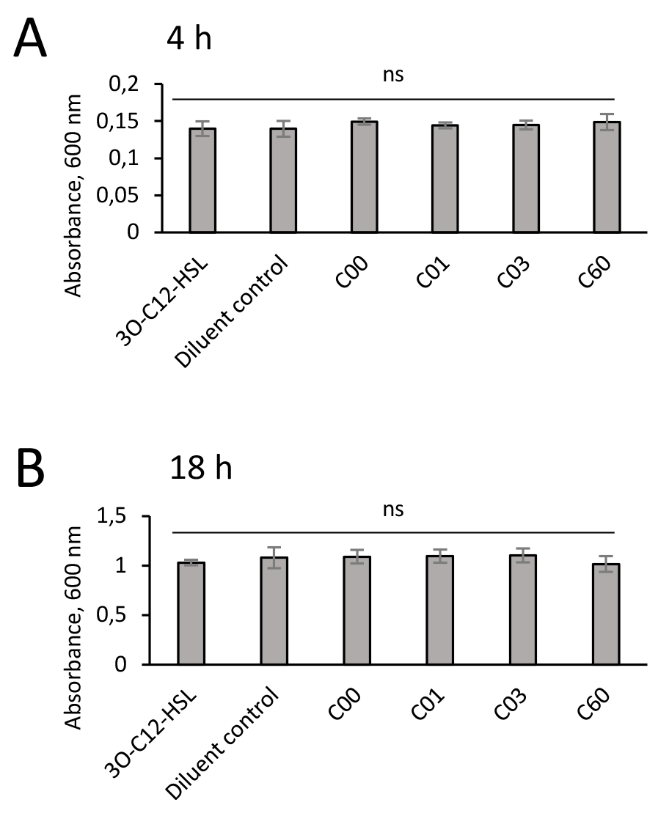
**

**Figure S1.** *P. aeruginosa* PA14 growth in the presence of AHL compounds. Bacteria were treated with acetonitrile (Diluent control) or exposed to 50 μM 3O-C_12_-HSL, or compound C00, C01, C03 or C60, and grown as a planktonic culture with good aeration. Bacterial growth was quantified in (**A**) 4-h and (**B**) 18-h cultures by measuring A600. At least 3 independent experiments in triplicates were performed on separate days on different bacteria cultures. Columns represent the means ± SE. None of the AHL compounds or 3O-C_12_-HSL were found to have any significant effect on the growth of the bacteria according to two-tailed Student’s *t*-test (ns).


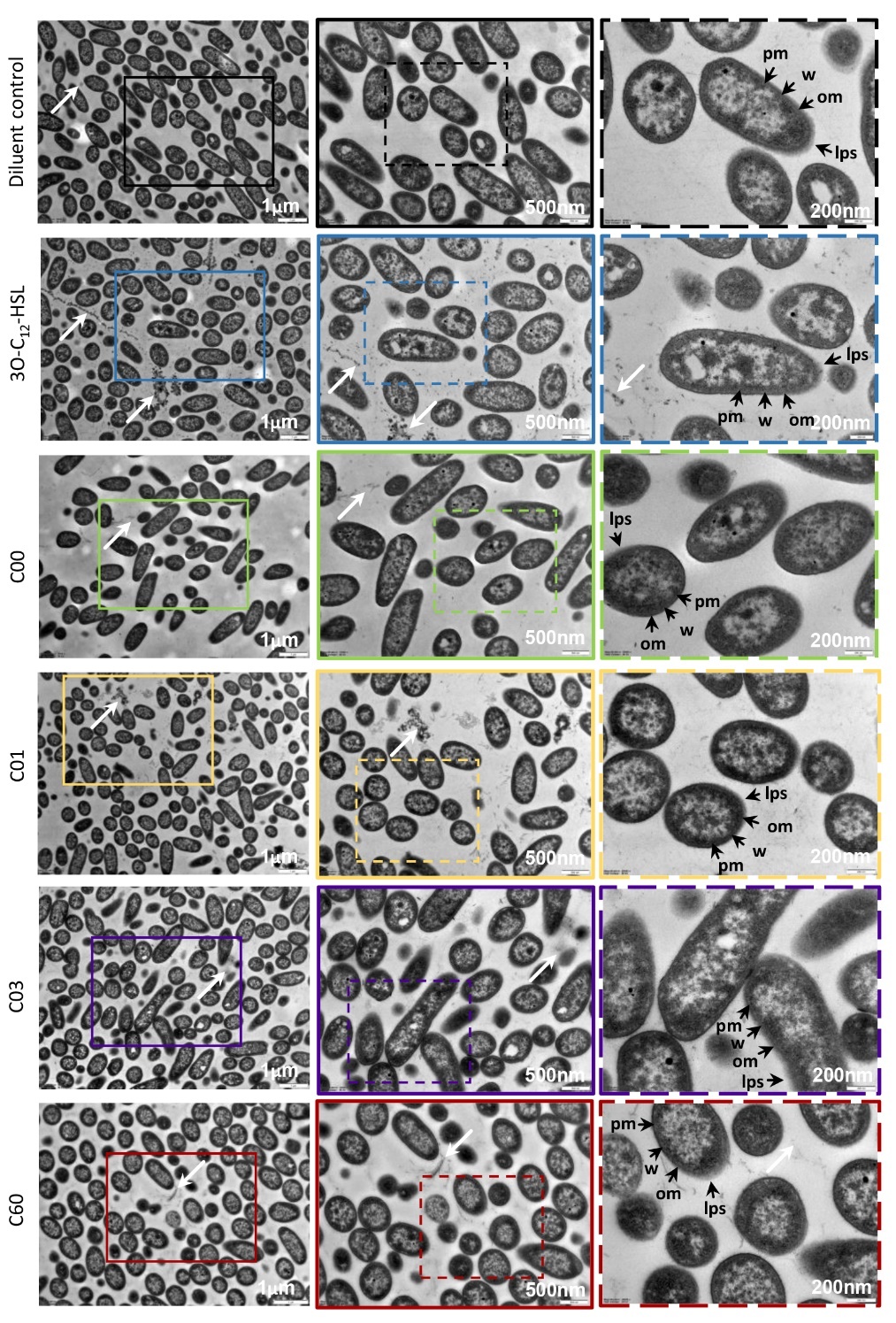


**Figure S2.** Ultrastructure of *P. aeruginosa* PA14 in response to AHL compounds. Bacteria were treated with acetonitrile (Diluent control) or exposed to 50 μM 3O-C_12_-HSL, or compound C00, C01, C03 or C60, and grown as a planktonic culture for 18 h. Cells were fixed, stained with uranyl acetate and analyzed by TEM. The data are from one of three independent experiments. Inserts with colored boards represent zoomed views at higher magnification. Left panel, bar 1 µm. Middle panel, bar 500 nm. Right panel, bar 200 nm. The cytoplasm and cytoplasmic components including nucleoid in bacteria can be distinguished respectively as less electron-dense (lighter) and comparatively higher electron-dense (darker) regions. Bacterial cells clearly showed plasma membrane (pm), cell wall (w), outer membrane (om) and lipopolysaccharide (LPS) around the bacteria (black arrows). Additional structures were observed between cells that visually resemble extracellular polymers or cell debris (white arrows).


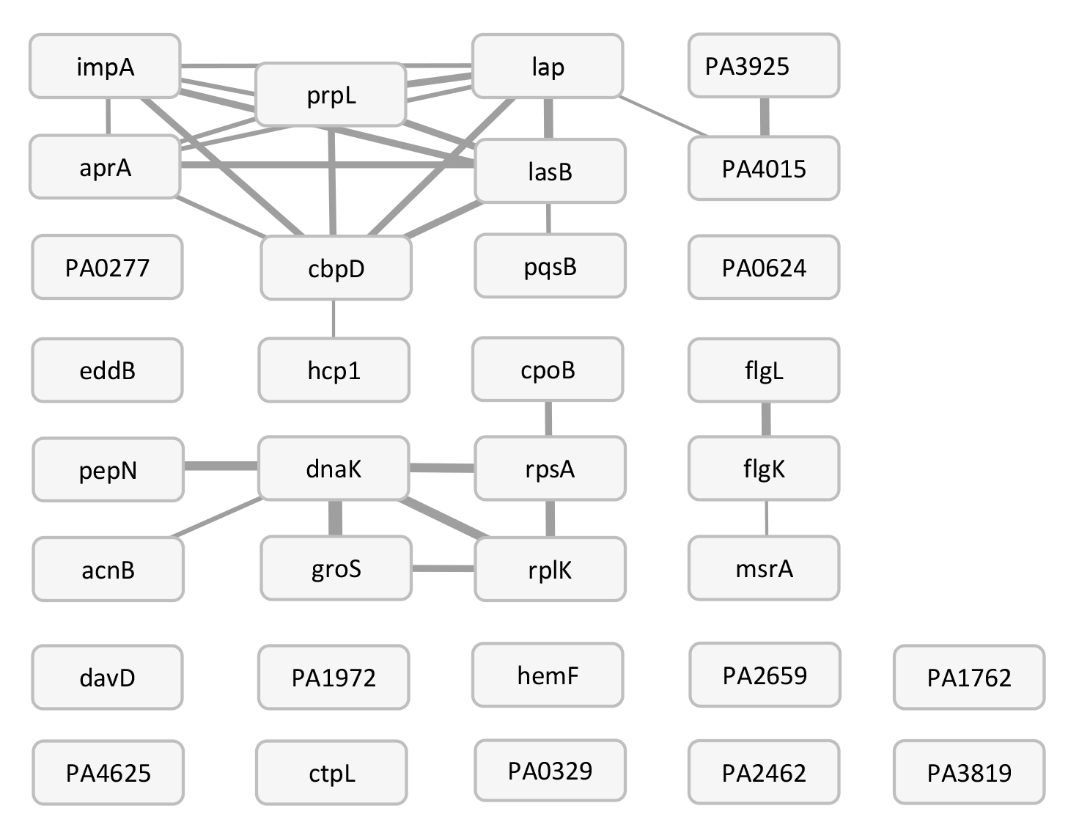


**Figure S3.** Bioinformatic assessment of potential interactions of the extracellular proteins with high expression levels in *P. aeruginosa* PA14 after 18-h treatment with 50 µM 3O-C_12_-HSL compared to the diluent control. The protein–protein interaction information was retrieved from Search Tool for the Retrieval of Interacting Genes and Proteins (STRING) ([Szklarczyk et al. 2019](#_ENREF_5)). Network nodes represent 33 upregulated proteins triggered by 3O-C_12_-HSL treatment as identified by MS-based relative quantification and presented in **Table 1**. Connections between nodes represent protein-protein interactions where line thickness indicates the strength of data support. Based on STRING and NCBI GO analyses the identified upregulated extracellular proteins were allocated to a functionally distinct groups, including toxic and degradative secreted enzymes; protein and DNA secretion system; synthesis of QS signals; flagellum-dependent cell motility and chemotaxis; adhesion; cell division and cell envelope synthesis; RNA and DNA processes; intracellular metabolism including cellular protein modification, folding processes, and iron homeostasis; virulence; biofilm formation (**Table 1**). Among these 33 upregulated proteins, at least 21 have been previously found to be QS-regulated, where most notable were: elastase B, protein hcp1, chitin-binding protein CpbD, 2-heptyl-4(1H)-quinolone synthase, aconitate hydratase B, glutarate-semialdehyde dehydrogenase, two aminopeptidases (lap and pepN), and two flagellar hook-associated proteins (flgL and flgK).

**
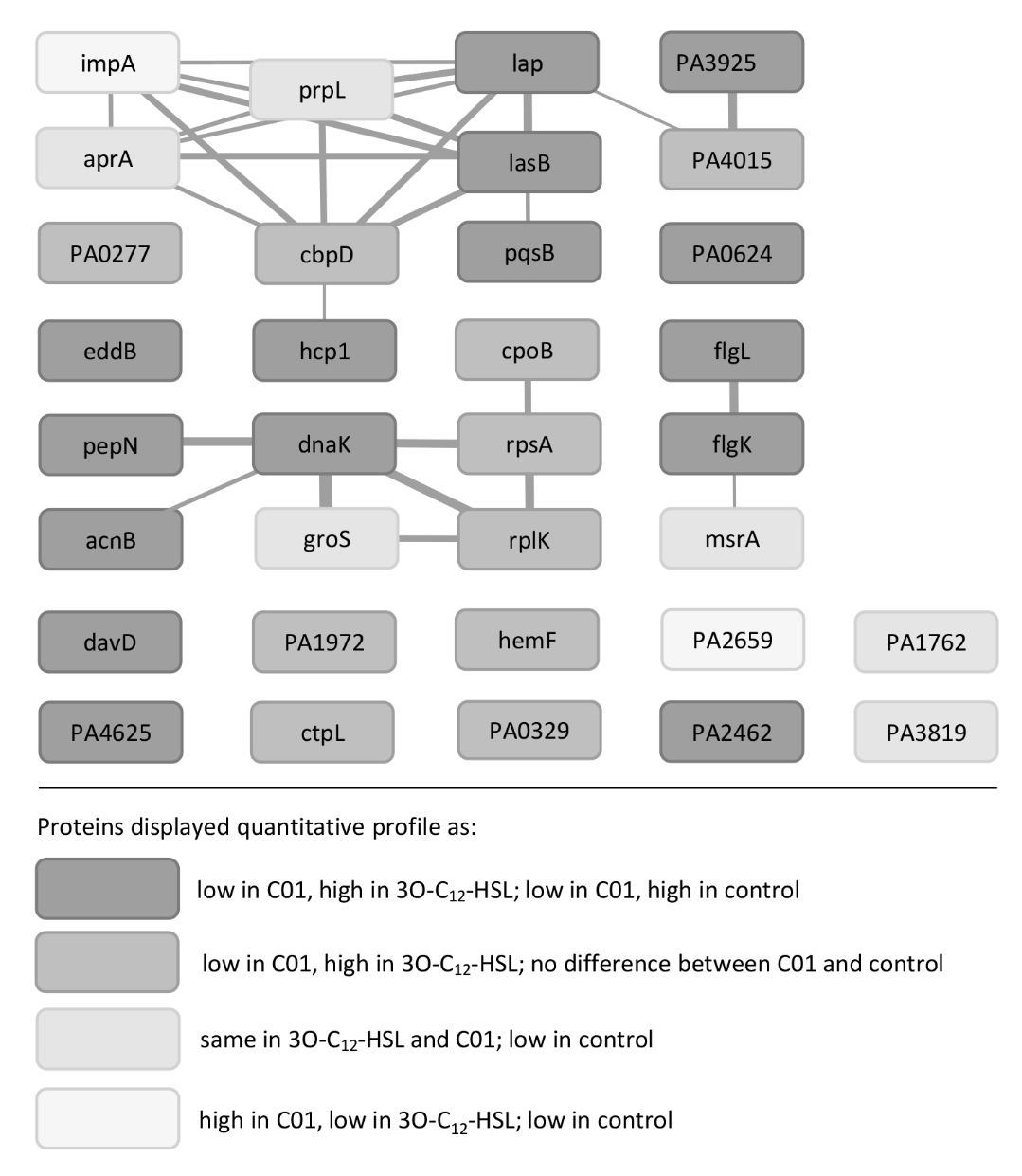
**

**Figure S4.** Bioinformatic assessment of interactome changes of the differentially expressed extracellular proteins in *P. aeruginosa* PA14 after 18-h treatment with 50 µM compound C01 or 3O-C_12_-HSL for 18 h compared to each other or to the diluent control. The protein–protein interaction information was retrieved from STRING. Network nodes represent 33 reference 3O-C_12_-HSL-responsive proteins (**Table 1**) selected for characterization of the *P. aeruginosa* excretome changes driven by treatment with compound C01 and shown in **Table 2**. The nodes are color-coded in four grey tones which illustrate identified proteins that displayed four different quantitative profiles as reflected in the lower panel of this figure. Connections between nodes represent potential protein-protein interactions where line thickness indicates the strength of data support.

| **A**  **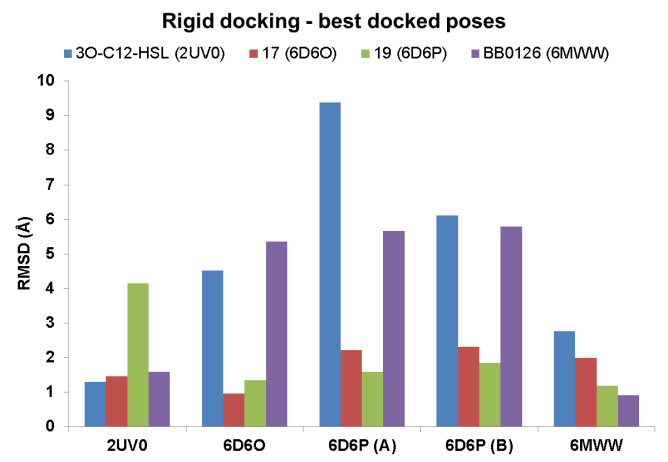** | **B**  **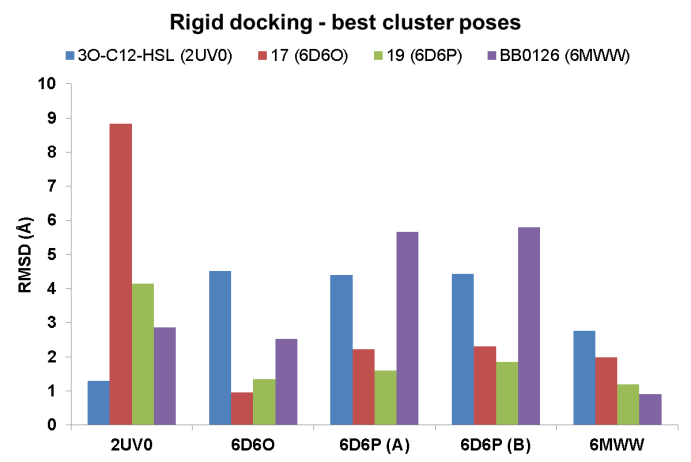** |
| --- | --- |
| **C**  **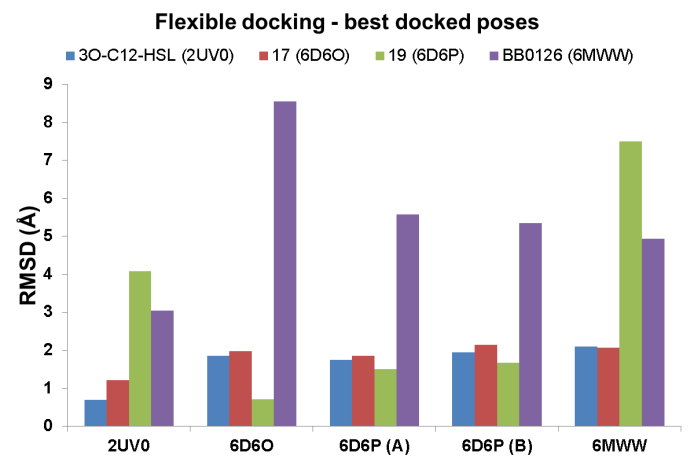** | **D**  **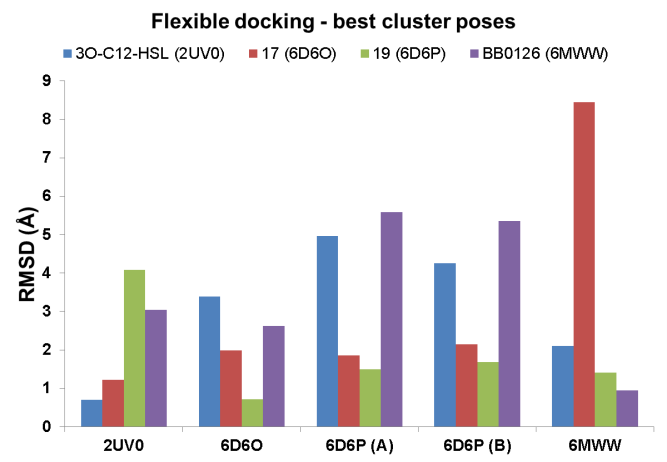** |
| **Figure S5.** Randomized conformation docking assessment. Root-mean-square deviation (RMSD) in Å between docked poses and experimental poses. Rigid docking: A) results from best-docked poses; B) results from best-cluster poses. Flexible docking: C) results from best-docked poses; D) results from best-cluster poses. Numerical values are reported in **Tables S5**, **S6**, **S7** and **S8** respectively. | |

| **A**  **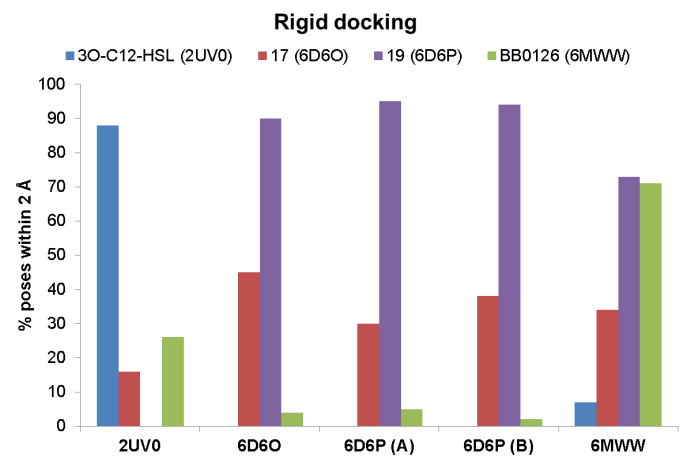** | **B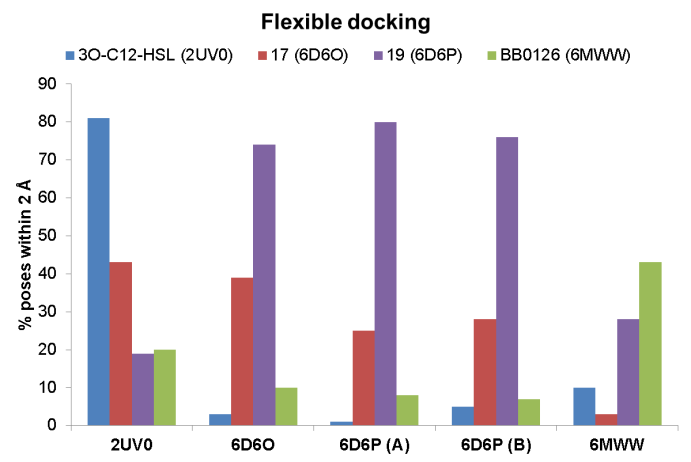** |
| --- | --- |
| **Figure S6.** Randomized conformation docking assessment. Percentage of docking poses showing a root-mean-square deviation (RMSD) within 2 Å from the experimental poses. A) results from rigid docking; B) results from flexible docking. Numerical values are reported in **Tables S9** and **S10**, respectively. | |

| **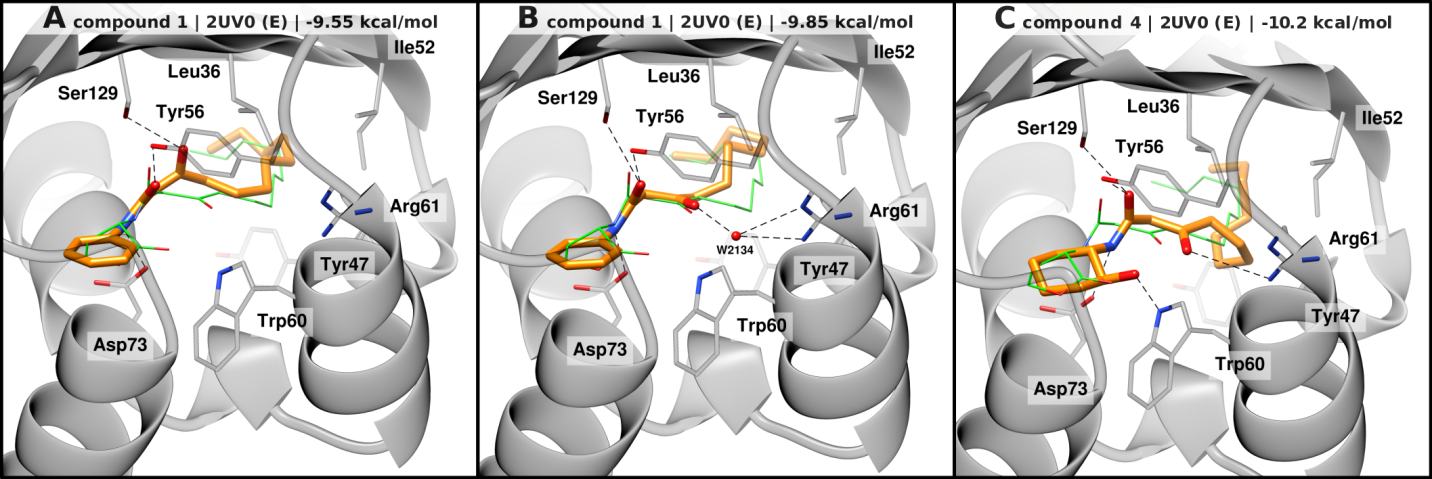** |
| --- |
| **Figure S7.** Binding mode prediction of AHL-like ligands, with modified head structures, in the ligand-binding pocket of LasR. Docking pose of the aniline analogue of 3O-C12-HSL, originally reported as compound 1:([Gerdt et al. 2014](#_ENREF_1)) A) without water; B) with water, ID 2134; C) docking pose of the 2-aminocyclohexanol analogue of 3O-C12-HSL, originally reported as compound 4.([Smith, Bu and Suga 2003](#_ENREF_4)) Compounds are shown as orange sticks. Protein structures and the native co-crystallized ligand are shown as gray cartoons and green wires, respectively. Selected side chains are shown as gray sticks. Putative hydrogen bonds are shown as black dashed lines. |

| **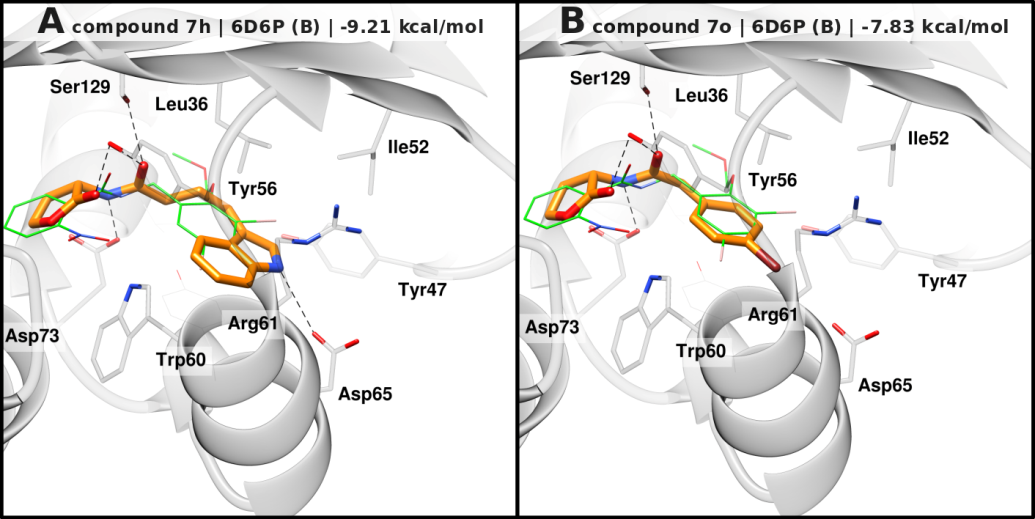** |
| --- |
| **Figure S8.** Predicted binding modes of AHL-like antagonists, containing bulky tail structures, in the ligand-binding pocket of LasR. Docking pose of A) compound 7h and B) compound 7o.([Geske et al. 2005](#_ENREF_2)) Compounds are shown as orange sticks. Protein structures and the native co-crystallized ligand are shown as gray cartoons and green wires, respectively. Selected side chains are shown as gray sticks. Putative hydrogen bonds are shown as black dashed lines. |

| **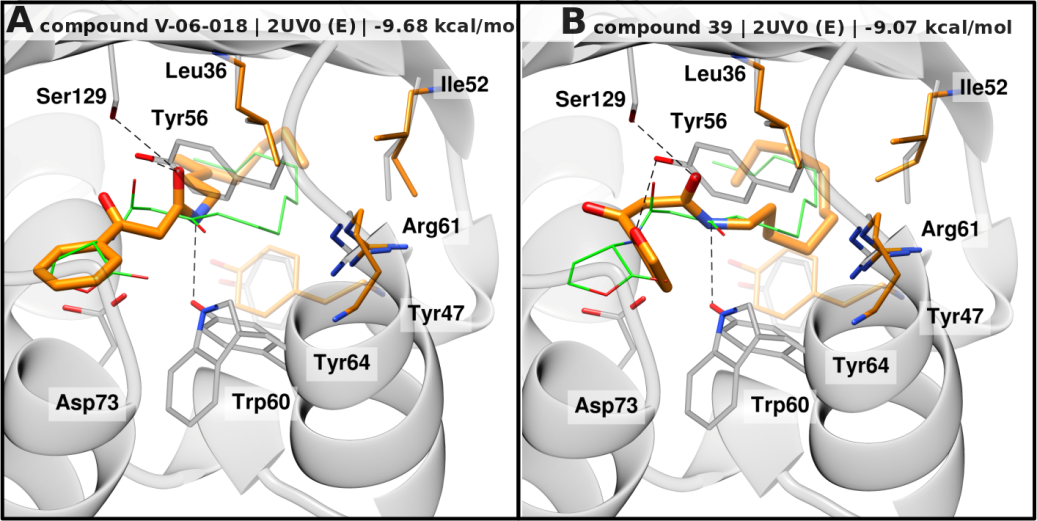** |
| --- |
| **Figure S9.** Predicted binding modes of non-native LasR antagonists in the ligand-binding pocket of LasR. Docking pose of A) compound V-06-18 and B) compound 39.([Manson et al. 2020](#_ENREF_3)) Compounds are shown as orange sticks. Protein structures and the native co-crystallized ligand are shown as gray cartoons and green wires, respectively. Selected side chains are shown as gray sticks; in the case of flexible docking, modelled side-chains of Leu36, Tyr47, Ile52 and Arg61 are shown as orange sticks.. Putative hydrogen bonds are shown as black dashed lines. |

**TABLES**

**Table S1.** Differentially expressed extracellular proteins in *P. aeruginosa* PA14 after treatment with 50 µM 3O-C_12_-HSL or compound C00 for 18 h compared to each other or to the diluent control.

| Identified proteins | Gene  Uniprot accession number | C00 compared to  3O-C_12_-HSL | | C00 compared to diluent control | |
| --- | --- | --- | --- | --- | --- |
|  |  | Fold change | *P*-value | Fold change | *P*-value |
|  |  |  |  |  |  |
| Proteins displayed quantitative profile as: low in C00, high in 3O-C_12_-HSL; low in C00, high in control | | | | | |
| Protein hcp1 | *hcp1* | 3.7 | < 0.00010 | 2.4 | 0.0042 |
| Flagellar hook-associated protein type 3 FlgL | *flgL* | 1.9 | < 0.00010 | 1.6 | < 0.00010 |
| Extracelullar DNA degradation protein EddB | *eddB* | 4.8 | < 0.00010 | 2.6 | 0.025 |
| 2-heptyl-4(1H)-quinolone synthase subunit PqsB | *pqsB* | INF | 0.00014 | INF | 0.019 |
| Flagellar hook-associated protein 1 | *flgK* | 1.8 | < 0.00010 | 1.6 | 0.00014 |
| Aminopeptidase | *lap* | 4.5 | < 0.00010 | 1.6 | 0.0083 |
| Haemagg act domain-containing protein | *PA4625* | 1.7 | < 0.00010 | 1.2 | 0.0088 |
| Cluster of Haemagg act domain-containing protein | *PA2462 PA0041* | 1 | < 0.00010 | 1 | 0.00066 |
| Methyl-accepting chemotaxis protein CtpL | *ctpL* | INF | 0.00082 | INF | 0.043 |
| Proteins displayed quantitative profile as: low in C00, high in 3O-C_12_-HSL; no difference between C00 and control | | | | | |
| Elastase | *lasB* | 1.1 | 0.0033 | 0.8 | 0.27 |
| Glutarate-semialdehyde dehydrogenase | *davD* | 1.7 | 0.0019 | 1.2 | 0.12 |
| Aminopeptidase N | *pepN* | 1.2 | 0.034 | 1 | 0.35 |
| Probable acyl-CoA thiolase | *PA3925* | 2.2 | 0.009 | 1.2 | 0.27 |
| Chitin-binding protein CbpD | *cbpD* | 1.1 | 0.00018 | 0.8 | 0.057 |
| Peptidase M48 domain-containing protein | *PA0277* | 6.7 | 0.015 | 1.3 | 0.61 |
| MaoC-like domain-containing protein | *PA4015* | 2.3 | 0.029 | 1 | 0.5 |
| Oxygen-dependent coproporphyrinogen-III oxidase | *hemF* | INF | 0.0027 | INF | 0.063 |
| UPF0339 protein PA0329 | *PA0329* | 1.8 | 0.021 | 1 | 0.47 |
| Phosphoethanolamine lipid A transferase | *PA1972* | INF | 0.00082 | INF | 0.063 |
| Proteins displayed the same quantitative profile in 3O-C_12_-HSL and C00; low in control | | | | | |
| Aconitate hydratase B | *acnB* | 0.7 | 0.3 | 0.6 | 0.0084 |
| Phage tail assembly protein | *PA0624* | 1.4 | 0.14 | 0.8 | 0.49 |
| Cell division coordinator CpoB | *cpoB* | 0.7 | 0.44 | 0.5 | 0.036 |
| 30S ribosomal protein S1 | *rpsA* | 1.2 | 0.22 | 0.6 | 0.25 |
| 50S ribosomal protein L11 | *rplK* | 1 | 0.31 | 0.6 | 0.086 |
| Lysyl endopeptidase | *prpL* | 0.9 | 0.14 | 0.7 | 0.0041 |
| Peptide methionine sulfoxide reductase MsrA | *msrA* | 2.7 | 0.1 | 0.5 | < 0.00010 |
| Peptidase C39 domain-containing protein | *PA1762* | 0.6 | 0.16 | 0.6 | 0.52 |
| Rick 17kDa anti domain-containing protein | *PA3819* | 0.8 | 0.53 | 0.5 | 0.038 |
| 10 kDa chaperonin | *groS* | 0.9 | 0.32 | 0.7 | 0.16 |
| Proteins displayed quantitative profile as: high in C00, low in 3O-C_12_-HSL; high in C00, low in control | | | | | |
| Chaperone protein DnaK | *dnaK* | 1 | 0.0016 | 1 | 0.043 |
| Serralysin | *aprA* | 0.7 | 0.036 | 0.5 | < 0.00010 |
| Immunomodulating metalloprotease | *impA* | 0.7 | 0.00035 | 0.6 | < 0.00010 |
| PepSY domain-containing protein | *PA2659* | 0.7 | 0.015 | 0.6 | < 0.00010 |

**Table S2**. Differentially expressed extracellular proteins in *P. aeruginosa* PA14 after treatment with 50 µM 3O-C_12_-HSL or compound C03 for 18 h compared to each other or to the diluent control.

| Identified proteins | Gene  Uniprot accession number | C03 compared to  3O-C_12_-HSL | | C03 compared to diluent control | |
| --- | --- | --- | --- | --- | --- |
|  |  | Fold change | *P*-value | Fold change | *P*-value |
|  |  |  |  |  |  |
| Proteins displayed quantitative profile as: low in C03, high in 3O-C_12_-HSL; low in C03, high in control | | | | | |
| Protein hcp1 | *hcp1* | 3.3 | 0.00011 | 2.1 | 0.024 |
| Flagellar hook-associated protein type 3 FlgL | *flgL* | 3.5 | < 0.00010 | 2.9 | < 0.00010 |
| Extracelullar DNA degradation protein EddB | *eddB* | INF | < 0.00010 | INF | 0.00019 |
| 2-heptyl-4(1H)-quinolone synthase subunit PqsB | *pqsB* | INF | 0.00031 | INF | 0.028 |
| Flagellar hook-associated protein 1 | *flgK* | 2.9 | < 0.00010 | 2.6 | < 0.00010 |
| Aminopeptidase | *lap* | 50 | < 0.00010 | 41 | < 0.00010 |
| Aminopeptidase N | *pepN* | 1.9 | 0.0013 | 1.5 | 0.042 |
| Cluster of Haemagg act domain-containing protein | *PA2462 PA0041* | 1.2 | < 0.00010 | 1.2 | 0.00042 |
| Chaperone protein DnaK | *dnaK* | 1.2 | < 0.00010 | 1.2 | 0.00096 |
| Proteins displayed quantitative profile as: low in C03, high in 3O-C_12_-HSL; no difference between C03 and control | | | | | |
| Glutarate-semialdehyde dehydrogenase | *davD* | 1.6 | 0.014 | 1.2 | 0.31 |
| Haemagg act domain-containing protein | *PA4625* | 1.7 | < 0.00010 | 1.2 | 0.068 |
| Probable acyl-CoA thiolase | *PA3925* | 2.9 | 0.0054 | 1.6 | 0.19 |
| Chitin-binding protein CbpD | *cbpD* | 1.2 | 0.00098 | 1.6 | 0.28 |
| Peptidase M48 domain-containing protein | *PA0277* | INF | 0.0045 | INF | 0.34 |
| MaoC-like domain-containing protein | *PA4015* | 3.8 | 0.011 | 1.7 | 0.3 |
| Oxygen-dependent coproporphyrinogen-III oxidase | *hemF* | INF | 0.0045 | INF | 0.083 |
| 30S ribosomal protein S1 | *rpsA* | 3.5 | 0.006 | 1.9 | 0.17 |
| UPF0339 protein PA0329 | *PA0329* | 3 | 0.0038 | 1.6 | 0.19 |
| Phosphoethanolamine lipid A transferase | *PA1972* | 8 | 0.0092 | 3 | 0.26 |
| Proteins displayed the same quantitative profile in 3O-C_12_-HSL and C03; low in control | | | | | |
| Elastase | *lasB* | 0.9 | 0.24 | 0.6 | < 0.00010 |
| Aconitate hydratase B | *acnB* | 0.9 | 0.49 | 0.7 | 0.041 |
| Cell division coordinator CpoB | *cpoB* | 0.6 | 0.11 | 0.4 | 0.0016 |
| 50S ribosomal protein L11 | *rplK* | 1.6 | 0.067 | 0.9 | 0.46 |
| Lysyl endopeptidase | *prpL* | 0.9 | 0.49 | 0.8 | 0.00013 |
| Serralysin | *aprA* | 0.9 | 0.47 | 0.7 | 0.011 |
| Peptide methionine sulfoxide reductase MsrA | *msrA* | 2.7 | 0.14 | 0.6 | 0.47 |
| Rick 17kDa anti domain-containing protein | *PA3819* | 1.2 | 0.26 | 0.8 | 0.18 |
| 10 kDa chaperonin | *groS* | 1.1 | 0.27 | 0.9 | 0.23 |
| Proteins displayed quantitative profile as: high in C03, low in 3O-C_12_-HSL; high in C03, low in control | | | | | |
| Immunomodulating metalloprotease | *impA* | 0.8 | 0.0089 | 0.7 | < 0.00010 |
| PepSY domain-containing protein | *PA2659* | 0.7 | < 0.00010 | 0.6 | < 0.00010 |
| Proteins displayed other quantitative profile | | | | | |
| Phage tail assembly protein | *PA0624* | 2.8 | 0.026 | 0.8 | 0.029 |
| Methyl-accepting chemotaxis protein CtpL | *ctpL* | 2.7 | 0.071 | 1.1 | 0.57 |
| Peptidase C39 domain-containing protein | *PA1762* | 4.7 | 0.09 | 1.3 | 0.65 |

**Table S3.** Differentially expressed extracellular proteins in *P. aeruginosa* PA14 after treatment with 50 µM 3O-C_12_-HSL or compound C60 for 18 h compared to each other or to the diluent control.

| Identified proteins | Gene  Uniprot accession number | C60 compared to 3O-C_12_-HSL | | C60 compared to diluent control | |
| --- | --- | --- | --- | --- | --- |
|  |  | Fold change | *P*-value | Fold change | *P*-value |
|  |  |  |  |  |  |
| Proteins displayed quantitative profile as: low in C60, high in 3O-C_12_-HSL; low in C60, high in control | | | | | |
| Protein hcp1 | *hcp1* | 3.3 | 0.00013 | 0.5 | 0.025 |
| Glutarate-semialdehyde dehydrogenase | *davD* | 2.4 | 0.00026 | 0.6 | 0.029 |
| Flagellar hook-associated protein type 3 FlgL | *flgL* | 3.2 | < 0.00010 | 0.4 | < 0.00010 |
| Extracelullar DNA degradation protein EddB | *eddB* | 9.7 | < 0.00010 | 0.2 | 0.0072 |
| 2-heptyl-4(1H)-quinolone synthase subunit PqsB | *pqsB* | INF | 0.00032 | 0 | 0.029 |
| Flagellar hook-associated protein 1 | *flgK* | 2.2 | < 0.00010 | 0.5 | < 0.00010 |
| Aminopeptidase | *lap* | 12 | < 0.00010 | 0.1 | < 0.00010 |
| Haemagg act domain-containing protein | *PA4625* | 2 | < 0.00010 | 0.7 | 0.0085 |
| Cluster of Haemagg act domain-containing protein | *PA2462 PA0041* | 1.4 | < 0.00010 | 0.8 | < 0.00010 |
| Chaperone protein DnaK | *dnaK* | 1.2 | < 0.00010 | 0.8 | 0.0013 |
| Proteins displayed quantitative profile as: low in C60, high in 3O-C_12_-HSL; no difference between C60 and control | | | | | |
| Aconitate hydratase B | *acnB* | 1.4 | 0.043 | 0.9 | 0.42 |
| Aminopeptidase N | *pepN* | 1.4 | 0.046 | 0.9 | 0.39 |
| Probable acyl-CoA thiolase | *PA3925* | 4.3 | 0.0009 | 0.4 | 0.062 |
| Peptidase M48 domain-containing protein | *PA0277* | INF | 0.0047 | 0 | 0.35 |
| Methyl-accepting chemotaxis protein CtpL | *ctpL* | 4 | 0.031 | 0.6 | 0.39 |
| Oxygen-dependent coproporphyrinogen-III oxidase | *hemF* | 6.7 | 0.024 | 0.3 | 0.26 |
| 30S ribosomal protein S1 | *rpsA* | 2.3 | 0.03 | 0.8 | 0.4 |
| UPF0339 protein PA0329 | *PA0329* | 2.6 | 0.0088 | 0.7 | 0.29 |
| 50S ribosomal protein L11 | *rplK* | 1.7 | 0.047 | 1 | 0.55 |
| Phosphoethanolamine lipid A transferase | *PA1972* | INF | 0.0016 | 0 | 0.084 |
| Peptidase C39 domain-containing protein | *PA1762* | INF | 0.023 | 0 | 0.35 |
| Proteins displayed the same quantitative profile in 3O-C_12_-HSL and C60 | | | | | |
| MaoC-like domain-containing protein | *PA4015* | 1.6 | 0.15 | 1.4 | 0.33 |
| Lysyl endopeptidase | *prpL* | 0.9 | 0.16 | 1.4 | < 0.00010 |
| Serralysin | *aprA* | 0.8 | 0.24 | 1.5 | 0.0014 |
| Peptide methionine sulfoxide reductase MsrA | *msrA* | 1.1 | 0.53 | 3.9 | 0.056 |
| Rick 17kDa anti domain-containing protein | *PA3819* | 1.1 | 0.38 | 1.4 | 0.098 |
| 10 kDa chaperonin | *groS* | 0.9 | 0.46 | 1.4 | 0.041 |
| Phage tail assembly protein | *PA0624* | 1.6 | 0.15 | 1.1 | 0.5 |
| Chitin-binding protein CbpD | *cbpD* | 1 | 0.36 | 1.5 | < 0.00010 |
| Cell division coordinator CpoB | *cpoB* | 0.7 | 0.3 | 2.2 | 0.015 |
| Proteins displayed quantitative profile as: high in C60, low in 3O-C_12_-HSL; high in C60, low in control | | | | | |
| Elastase | *lasB* | 0.7 | 0.00014 | 2.1 | < 0.00010 |
| Immunomodulating metalloprotease | *impA* | 0.8 | 0.0026 | 1.4 | < 0.00010 |
| PepSY domain-containing protein | *PA2659* | 0.7 | 0.00036 | 1.6 | < 0.00010 |

| **Table S4.** LasR-agonist structures that were selected for docking calculations. | |
| --- | --- |
| **PDB ID** | **Chain** |
| 2UV0^a^ | E |
| 6D6O | B |
| 6MWW | B |
| 6D6P^b^ | A |
| 6D6P^b^ | B |
| ^a^ Self-redocking experiments were performed either removing or maintaining a bridging water molecule (ID 2134 in pdb)  ^b^ 6D6P shows two different orientations of Tyr93 which are represented in chains A and B, respectively. | |

| **Table S5.** Rigid docking, randomized conformation docking assessment. Root-mean-square deviation (RMSD) in Å between best-docked poses and experimental poses. Results from re-docking are shown in bold. | | | | | | |
| --- | --- | --- | --- | --- | --- | --- |
| **LIGAND** | **TARGET** | | | | | |
|  | **2UV0** | **2UVO wat** | **6D6O** | **6D6P (A)** | **6D6P (B)** | **6MWW** |
| **3O-C12-HSL (2UV0)** | **1.29** | **0.77** | 4.52 | 9.38 | 6.11 | 2.77 |
| **17 (6D6O)** | 1.46 | - | **0.96** | 2.22 | 2.31 | 1.99 |
| **19 (6D6P)** | 4.15 | - | 1.35 | **1.59** | **1.85** | 1.19 |
| **BB0126 (6MWW)** | 1.59 | - | 5.35 | 5.66 | 5.79 | **0.91** |

| **Table S6.** Rigid docking, randomized conformation docking assessment. Root-mean-square deviation (RMSD) in Å between best-cluster poses and experimental poses. Results from re-docking are shown in bold. | | | | | | |
| --- | --- | --- | --- | --- | --- | --- |
| **LIGAND** | **TARGET** | | | | | |
|  | **2UV0** | **2UVO wat** | **6D6O** | **6D6P (A)** | **6D6P (B)** | **6MWW** |
| **3O-C12-HSL (2UV0)** | **1.29** | **0.77** | 4.52 | 4.4 | 4.43 | 2.77 |
| **17 (6D6O)** | 8.84 | - | **0.96** | 2.22 | 2.31 | 1.99 |
| **19 (6D6P)** | 4.15 | - | 1.35 | **1.59** | **1.85** | 1.19 |
| **BB0126 (6MWW)** | 2.86 | - | 2.52 | 5.66 | 5.79 | **0.91** |

| **Table S7.** Flexible docking, randomized conformation docking assessment. Root-mean-square deviation (RMSD) in Å between best docked poses and experimental poses. Results from re-docking are shown in bold. | | | | | | |
| --- | --- | --- | --- | --- | --- | --- |
| **LIGAND** | **TARGET** | | | | | |
|  | **2UV0** | **2UVO wat** | **6D6O** | **6D6P (A)** | **6D6P (B)** | **6MWW** |
| **3O-C12-HSL (2UV0)** | **0.7** | **1.12** | 1.86 | 1.75 | 1.95 | 2.1 |
| **17 (6D6O)** | 1.22 | - | **1.98** | 1.85 | 2.15 | 2.07 |
| **19 (6D6P)** | 4.08 | - | 0.71 | **1.5** | **1.68** | 7.5 |
| **BB0126 (6MWW)** | 3.04 | - | 8.56 | 5.58 | 5.35 | **4.94** |

| **Table S8.** Flexible docking, randomized conformation docking assessment. Root-mean-square deviation (RMSD) in Å between best cluster poses and experimental poses. Results from re-docking are shown in bold. | | | | | | |
| --- | --- | --- | --- | --- | --- | --- |
| **LIGAND** | **TARGET** | | | | | |
|  | **2UV0** | **2UVO wat** | **6D6O** | **6D6P (A)** | **6D6P (B)** | **6MWW** |
| **3O-C12-HSL (2UV0)** | **0.7** | **1.12** | 3.39 | 4.96 | 4.25 | 2.1 |
| **17 (6D6O)** | 1.22 | - | **1.98** | 1.85 | 2.15 | 8.45 |
| **19 (6D6P)** | 4.08 | - | 0.71 | **1.5** | **1.68** | 1.41 |
| **BB0126 (6MWW)** | 3.04 | - | 2.63 | 5.58 | 5.35 | **0.95** |

| **Table S9.** Rigid docking, randomized conformation docking assessment. Percentage of docking poses showing a root-mean-square deviation (RMSD) within 2 Å from the experimental poses. Results from re-docking are shown in bold. | | | | | |
| --- | --- | --- | --- | --- | --- |
| **LIGAND** | **TARGET** | | | | |
|  | **2UV0** | **6D6O** | **6D6P (A)** | **6D6P (B)** | **6MWW** |
| **3O-C12-HSL (2UV0)** | **88** | 0 | 0 | 0 | 7 |
| **17 (6D6O)** | 16 | **45** | 30 | 38 | 34 |
| **19 (6D6P)** | 0 | 90 | **95** | **94** | 73 |
| **BB0126 (6MWW)** | 26 | 4 | 5 | 2 | **71** |

| **Table S10.** Flexible docking, randomized conformation docking assessment. Percentage of docking poses showing a root-mean-square deviation (RMSD) within 2 Å from the experimental poses. Results from re-docking are shown in bold. | | | | | |
| --- | --- | --- | --- | --- | --- |
| **LIGAND** | **TARGET** | | | | |
|  | **2UV0** | **6D6O** | **6D6P (A)** | **6D6P (B)** | **6MWW** |
| **3O-C12-HSL (2UV0)** | **81** | 3 | 1 | 5 | 10 |
| **17 (6D6O)** | 43 | **39** | 25 | 28 | 3 |
| **19 (6D6P)** | 19 | 74 | **80** | **76** | 28 |
| **BB0126 (6MWW)** | 20 | 10 | 8 | 7 | **43** |

**References**

Gerdt, J. P., C. E. McInnis, T. L. Schell, F. M. Rossi & H. E. Blackwell (2014) Mutational analysis of the quorum-sensing receptor LasR reveals interactions that govern activation and inhibition by nonlactone ligands. *Chem Biol,* 21**,** 1361-1369.

Geske, G. D., R. J. Wezeman, A. P. Siegel & H. E. Blackwell (2005) Small molecule inhibitors of bacterial quorum sensing and biofilm formation. *J Am Chem Soc,* 127**,** 12762-3.

Manson, D. E., M. C. O'Reilly, K. E. Nyffeler & H. E. Blackwell (2020) Design, Synthesis, and Biochemical Characterization of Non-Native Antagonists of the Pseudomonas aeruginosa Quorum Sensing Receptor LasR with Nanomolar IC(50) Values. *ACS Infect Dis,* 6**,** 649-661.

Smith, K. M., Y. Bu & H. Suga (2003) Library screening for synthetic agonists and antagonists of a Pseudomonas aeruginosa autoinducer. *Chem Biol,* 10**,** 563-71.

Szklarczyk, D., A. L. Gable, D. Lyon, A. Junge, S. Wyder, J. Huerta-Cepas, M. Simonovic, N. T. Doncheva, J. H. Morris, P. Bork, L. J. Jensen & C. V. Mering (2019) STRING v11: protein-protein association networks with increased coverage, supporting functional discovery in genome-wide experimental datasets. *Nucleic Acids Res,* 47**,** D607-D613.
